# Immunodominant *Mycobacterium tuberculosis* Protein Rv1507A Elicits Th1 Response and Modulates Host Macrophage Effector Functions

**DOI:** 10.3389/fimmu.2020.01199

**Published:** 2020-07-21

**Authors:** Simran Kaur Arora, Anwar Alam, Nilofer Naqvi, Javeed Ahmad, Javaid Ahmad Sheikh, Syed Asad Rahman, Seyed Ehtesham Hasnain, Nasreen Zafar Ehtesham

**Affiliations:** ^1^Institute of Molecular Medicine, Jamia Hamdard, New Delhi, India; ^2^ICMR-National Institute of Pathology, New Delhi, India; ^3^Department of Biotechnology, Jamia Hamdard, New Delhi, India; ^4^BioInception Pvt. Ltd., Chelmsford, United Kingdom; ^5^Dr. Reddy's Institute of Life Sciences, Hyderabad, India

**Keywords:** CD4^+^/CD8^+^ T cells, central memory, effector memory, *Mycobacterium smegmatis* knock-in, TB subunit vaccine

## Abstract

*Mycobacterium tuberculosis* (*M. tb*) persists as latent infection in nearly a quarter of the global population and remains the leading cause of death among infectious diseases. While BCG is the only vaccine for TB, its inability to provide complete protection makes it imperative to engineer BCG such that it expresses immunodominant antigens that can enhance its protective potential. *In-silico* comparative genomic analysis of Mycobacterium species identified *M. tb* Rv1507A as a “signature protein” found exclusively in *M. tb*. *In-vitro* (cell lines) and *in-vivo* experiments carried out in mice, using purified recombinant Rv1507A revealed it to be a pro-inflammatory molecule, eliciting significantly high levels of IL-6, TNF-α, and IL-12. There was increased expression of activation markers CD69, CD80, CD86, antigen presentation molecules (MHC I/MHCII), and associated Th1 type of immune response. Rv1507A knocked-in *M. smegmatis* also induced significantly higher pro-inflammatory Th1 response and higher survivability under stress conditions, both *in-vitro* (macrophage RAW264.7 cells) and *in-vivo* (mice). Sera derived from human TB patients showed significantly enhanced B-cell response against *M. tb* Rv1507A. The ability of *M. tb* Rv1507A to induce immuno-modulatory effect, B cell response, and significant memory response, renders it a putative vaccine candidate that demands further exploration.

## Introduction

*Mycobacterium tuberculosis* (*M. tb*), the intracellular pathogen causing Tuberculosis (TB), still cause nearly 10 million new TB cases annually and about 1.45 million deaths were reported in 2018 alone (1). It is therefore imperative that prevention strategies such as development of new vaccines against TB be undertaken on priority. Bacillus Calmette Guerin (BCG) vaccine provides a degree of immunity against *M. tb* infections in children, however its efficacy is variable among adults populations due to its inability to mount a strong and sustained memory response (2). Development of new TB vaccine or improving the efficacy of BCG is therefore important to achieve the goals laid down in END-TB strategy (3). The emerging role of trained immunity and enhanced efficacy of BCG by altering the vaccination route has reinvigorated the interest toward live vaccines (4–7). Recombinant BCG vaccines like rBCG30, BCG expressing RD1 proteins, rBCG ΔureC::hly (VPM1002), etc. have been found to provide enhanced protection as compared to wild type BCG (8, 9). Recombinant *M. smegmatis* based vaccines have been also shown to induce effective immunity against *M. tb* with considerable protective potential (10–13). Exploratory studies to identify antigens that can enhance the potency of BCG vaccine are exigently warranted.

*M. tb* primarily infects and resides within macrophages. The phagocytozed *M. tb* can escape phago-lysosomal fusion, avoid lysis within macrophage, and modulate the activity of other immune cells by regulating cytokine secretion (14). *M. tb* also impairs the function of APCs by modulation of antigen presentation or expression of co-stimulatory molecules (15, 16). Cell-mediated immunity regulated by Th1 cytokines is considered important to eradicate the intracellular pathogens while Th2 response has been found to have important role in clearing the extracellular infectious agents (17). Although some vaccines do suggest a role of Th17 cells in protection particularly during mucosal administration, the exact role is still controversial (18–21). Increased focus on mucosal based vaccines in recent years suggest that IL-17A responses may be necessary for effective vaccination-induced anti-mycobacterial immunity in the lung (22). Th1 cytokine IFN-γ helps in activation of macrophages and phagolysosome formation that lead to production of reactive oxygen and nitrogen species, resulting in elimination of infection (23). TNF-α works in tandem with IFN-γ and helps in activation of macrophages and in production of reactive oxygen and nitrogen species, thereby mediating the bacteriostatic function of macrophages (24, 25). IFN-γ and TNF-α knocked out mice were found to be more susceptible to *M. tb* infection (26). Therefore, vaccines or protein antigens that boost Th1 immune response will likely be more effective to control *M. tb* infection, though this is not the only protective signature (27, 28). Moreover, despite evoking Th1 response, some vaccines were nevertheless ineffective, demanding exploratory vaccine studies to predict novel biomarkers of protection (29).

Studies to decipher the role of genes and their encoded proteins that aid in virulence of *M. tb* have been at the center stage of research. Numerous efforts have been made to unravel the immunomodulatory effect of mycobacterial proteins to gain insights into pathogenesis or development of novel vaccine candidates (30–32). Many of the proteins are known to activate cell mediated Th1 type host immune responses (33–35). Several other proteins have also been unveiled that hamper the immune responses and play a role in pathogenesis (36–40). Numerous attempts have been made to incorporate *M. tb* proteins into non-pathogens to enhance their vaccine potential (41). Though the strategy is feasible, evidence of reversion to virulence has also been reported (42). Thus, candidate proteins need to be examined, *in-vitro* as well as *in-vivo*, for further exploration as a vaccine candidate. We earlier reported a comparative genomic and proteomic analysis of several opportunistic, non-pathogenic and pathogenic species of mycobacteria to show that *M. tb* evolved from non-pathogenic soil-dwelling bacteria through genome reduction (43). In this process, several key genes that were essential for survival and virulence of *M. tb* were either retained or expanded (44). Elucidating the function and immunological significance of gene products, present in *M. tb* but absent in non-pathogenic mycobacterial species, are therefore important. Taking clue from these studies we identified *Rv1507A* gene, the so-called “signature protein” present exclusively in *M. tb*. This gene is present in RD4 region of *M. tb* affirming its absence in BCG. Our analysis also revealed its exclusive presence in *M. tb* and absence from all mycobacterial species studied therein. We describe immunological and other attributes of this otherwise hypothetical protein in terms of its likely utility as a vaccine candidate that demands further exploration.

## Materials and Methods

### Reagents and Cell Culture

*Mycobacterium smegmatis* mc^2^155, obtained from ATCC (Virginia, United States), was maintained as glycerol stocks in our laboratory. Middlebrook 7H9 growth media supplemented with 10% OADC (Himedia Laboratories, Mumbai, India) was used to subculture bacterial strains. DMEM supplemented with 10% Fetal Bovine Serum (FBS), hereafter called complete media, was used for sub culturing RAW264.7 cells. Isopropyl β-D-1-thiogalactopyranoside (IPTG), Sarcosyl, imidazole, kanamycin, Staurosporine, and MTT were obtained from Merck Limited, Mumbai, India. All cell culture reagents including DMEM were procured from Gibco (Thermo Fisher Scientific India Pvt Ltd, Mumbai, India). Antibodies, Middlebrook 7H11 agar, Middlebrook 7H9 agar, and 7H10 media were obtained from BD Biosciences (San Jose, CA, USA). Enzymes, ELISA kit, and toxicity removal kit were obtained from NEB (Massachusetts, USA), PeproTech US (Rocky Hill, NJ, USA), and Norgen (Thorold, ON, Canada), respectively, or otherwise mentioned. All reagents used in our experiments were of analytical grade.

### *In-silico* Structural and Functional Analyses of Rv1507A Protein

The prediction of protein binding sites in disordered regions was done using Anchor (https://iupred2a.elte.hu) and T cell/B cell epitope prediction was done by IEDB (http://tools.immuneepitope.org/) tool. The subcellular localization and secretory nature of this protein was determined by PredictProtein (https://www.predictprotein.org/).

### Cloning, Expression, Purification of *M. tb* Rv1507A, and Generation of *M. smegmatis* Knock-in

In order to evaluate the secretory nature Rv1507A, the presence of this protein in culture filtrate of *M. tb* was evaluated by SDS-PAGE analysis. The gel was blotted onto PVDF membrane and probed with polyclonal sera raised against Rv1507A. The ORF encoding *M. tb Rv1507A* gene was amplified using polymerase chain reaction (PCR) using forward and reverse primers generated from *M. tb* H_37_Rv genomic database (Supplementary Table 1). *Rv1507A* was inserted in pET28a expression vector (Addgene, Massachusetts, USA) using *Eco*RI and *Hin*dIII restriction sites to construct plasmid pET28a_Rv1507A. *E.coli* BL21(DE3) expression strain was transformed with recombinant constructs and the culture was incubated with 1 mM IPTG for 3 h at 37°C to induce expression of recombinant protein, Rv1507A, which was purified using Ni-NTA affinity column (Qiagen, Hilden, Germany) and eluted with 300 mM imidazole (45). The dialyzed protein was concentrated using centricons (Merck Limited, Mumbai, India) with 3 kDa cut off, treated with Polymyxin B beads (Merck Limited, Mumbai, India) to remove bacterial endotoxin contamination and examined using SDS-PAGE.

Mycobacterial integration expression vector pST-Ki was used to sub clone *Rv1507A* gene from pET28a_Rv1507A construct to create pST-Ki_Rv1507A plasmid, as described previously (46). Electroporation (Bio-Rad laboratories, California, United States) of pST-Ki_Rv1507A into *M. smegmatis* was carried out to generate *M. smegmatis* Rv1507A knock-in. Kanamycin (50 μg/ml) containing Middlebrook 7H11 agar plates supplemented with 0.5% glycerol were used to select the positive colonies and confirmed through PCR amplification using the standard protocols, as described previously (40). The integration of pST-Ki_Rv1507A into the genome of *M. smegmatis* was confirmed by a three step sequential process. Firstly, *M. smegmatis* positive colonies harboring Rv1507A (Ms_Rv1507A) and vector pST-Ki (Ms_Vc) were grown on kanamycin containing 7H11 agar plates. Positive colonies were selected and consecutively passaged for seven generations on antibiotic containing plates. In the second step, the selected colonies were plated on kanamycin negative plate and passaged for five generations. Lastly, the integration of cassette was confirmed by again plating on kanamycin plate and passaged for seven generations. Confirmed colonies were grown till log phase, harvested, centrifuged and the pellet was heated at 95°C for 30 min after dissolving in SDS-PAGE loading dye. Lysate fractions were centrifuged at 13,000 rpm for 10 min and the supernatant was loaded on 10% Tricine gel. Rv1507A protein was confirmed by western blotting, using anti-rabbit polyclonal Rv1507A antibody generated in rabbit (described below). The blots were visualized after incubation with HRP labeled anti-rabbit IgG antibody.

### Immunization of Mice

All experiments using lab animals were conducted as per the guidelines provided by the Committee for the Purpose of Control and Supervision on Experiments on Animals (CPCSEA) (www.envfor.nic.in/divisions/awd/ cpcsea_laboratory.pdf) and in compliance with the protocols approved by Institutional Biosafety Committee and Institutional Animal Ethics Committee, National Institute of Pathology, New Delhi, India (Approval No. NIP/IAEC-1701). All animals were housed in positive-pressure units under ambient conditions (25°C, 12 h light/dark cycle). Purified recombinant protein *M. tb* Rv1507A (200 μg/ml), emulsified with an equal volume of Freund's incomplete adjuvant (Merck Limited, Mumbai, India), was injected subcutaneously in rabbits at two different sites (0.5 ml/site) followed by two booster immunizations (100 μg/ml) with Freund's incomplete adjuvant at 15 day intervals. Titer of polyclonal antibodies against *M. tb* Rv1507A in rabbit sera was determined by dot-blot technique 2 weeks after final immunization.

Inbred BALB/c mice (Female, 8–12 week, 20–25 g) were obtained from the National Institute of Immunology (New Delhi, India). Primary immunization was carried out with purified recombinant *M. tb* Rv1507A protein (10 μg) in 100 μl PBS buffer, administered subcutaneously. We avoided use of adjuvant to minimize the immunomodulatory bias introduced by use of adjuvants (47, 48). A booster dose of this protein (10 μg in 100 μl PBS) was administered on 10th day of immunization followed by another booster after 10 days. Control animals were injected with equal amount of PBS at the immunizing site. Thirty days after primary immunization, mice were sacrificed to isolate splenocytes for other functional assays. Thereafter, in a separate set of experiments, BALB/c mice (female, 8–12 week, 20–25g) were obtained from National Institute of Biologicals (Noida, India) and randomized into three groups (*n* = 6/group). Mice were intra-peritoneally injected with PBS, Ms_Vc, or Ms_Rv1507A (1 × 10^7^ CFU), respectively, to evaluate the antigenicity and immunogenicity of this protein (49–51). Groups of mice were also infected intra-tracheally to mimic aerogenic infection and gross pathology of lungs was observed as explained in the following. Animals were randomized and treated similarly as discussed above for intra-peritoneal infections except bacteria were delivered via intra-tracheal instillation directly to lung surface.

### Human Subjects

All experiments involving samples from human subjects were approved by Institutional Ethics Committee (IEC), National Institute of Pathology, New Delhi, India. Informed consent was obtained from patient and healthy individuals (controls) included in the study. Blood samples were collected from treatment naïve, fresh pulmonary TB patients (*n* = 31) and healthy individuals (*n* = 19) from All India Institute of Medical Sciences (AIIMS), New Delhi, India. Diagnosis of TB was primarily based on microscopic examination of presence of acid-fast bacilli in sputum smear and chest radiography of patients. Healthy individuals with unknown TST/IGRA status were included as controls. Considering 40% of the Indian population to be latently infected (52), we assume about 8 individuals out of 19 controls to be latently infected. Exclusion criteria in both groups included HIV or HBsAg positivity along with any co-morbid disease. Healthy controls enrolled in study had no known history of any contact with TB patients or samples.

### Splenocyte Isolation

Mice were sacrificed after 30 days of primary immunization. Splenomegaly was evident in animals infected with Ms_Rv1507A. For *in-vitro* assays, spleen cells from BALB/c mice were obtained using standard protocols (45). Briefly, spleen was isolated, crushed gently, and perfused using 26-gauge needle. The cell suspension was then passed through a cell strainer, centrifuged, and resuspended in RBC lysis buffer (0.84% NH_4_Cl solution) for a minute. The final clean preparation of splenocytes, devoid of erythrocytes, were centrifuged and re-suspended in complete media. Splenocytes (1 × 10^6^) from all the groups were re-stimulated with recombinant protein (10 μg/ml) and cultured for 48 h. The supernatant was collected and stored for further analysis.

### Estimation of IgG Level Against Rv1507A in Mice and Human Sera

Estimation of IgG response in mice and reactivity of human sera against Rv1507A was assessed using ELISA (53). Briefly, 96 well-plates were coated with purified recombinant Rv1507A protein (10 μg/ml) and kept at 4°C overnight. The plates were thrice washed with PBST (1X PBS pH 7.2, 0.05% tween20) and then blocked with 10% FBS for 1 h at room temperature. Serum samples, obtained from mice immunized with Rv1507A protein and from mice immunized with Ms_Vc or Ms_Rv1507A, were added at 1:100 dilution to each well and incubated for 2 h. Goat anti-mouse IgG-HRP secondary antibody (Merck Limited, Mumbai, India) was added at 1:10000 dilution for 1 h (54). After five washes, TMB substrate was added, reaction was stopped with 2N H_2_SO_4_, and absorbance was measured at 450 nm in a spectrophotometer to calculate the amount of serum IgG level. To assess the specificity of antibodies in patient sera against Rv1507A, the protein-coated plates were incubated with patient serum samples at pre-standardized 1:200 dilutions that provides optimal signal: noise ratio. HRP conjugated secondary antibody was added at 1:10000 dilution. SIGMAFAST^TM^ OPD tablets were used as a substrate. The reaction was finally stopped by adding 50 μl of 3N H_2_SO_4_. The optical density was measured at a wavelength of 492 nm. Diagnostic values of IgG responses was examined by analysis of Receiver-operating characteristic (ROC) curve to determine the test performance in terms of sensitivity and specificity.

### Estimation of Cytokine Levels

RAW264.7 cells were cultured with different concentrations of Rv1507A protein. Additionally, splenocytes from mice infected with Ms_Vc or Ms_Rv1507A (0.1 × 10^6^ cells/well) were also seeded in 96-well plate, stimulated with different concentration of recombinant protein Rv1507A and incubated at 37°C for 12, 24, and 48 h. The levels of secreted cytokines TNF-α, IL-6, and IL-12, were quantified using murine standard ELISA Development Kit (PeproTech, Rocky Hill, NJ, USA) as per the manufacturer protocol. Splenocytes from mice immunized with recombinant Rv1507A were *in-vitro* cultured and stimulated and IFNγ was evaluated in culture supernatants by ELISA. Briefly, 96 well ELISA plates were coated with 100 μl of capture antibody and incubated at room temperature overnight. Plates were washed with 300 μl PBST and treated with 1% BSA, used as blocking buffer. After 1.5 h of blocking, the plates were again washed with PBST and 100 μl of supernatant were added for 2 h. After 5 washes with PBST, 100 μl of detection antibody was added followed by addition of enzyme-avidin HRP conjugate (100 μl/well) and TMB substrate (100 μl/well) for color development. The reaction was stopped using 2N H_2_SO_4_ and absorbance observed at 450 nm and reference wavelength at 570 nm.

### Measurement of ROS, NO Levels, and Apoptosis in Macrophage

RAW 264.7 cells (2 × 10^5^) were infected with Ms_Rv1507A or Ms_Vc at MOI 1:10 in incomplete DMEM for 4 h at 37°C. Cells were washed thrice with PBS and incubated in complete media supplemented with gentamycin. After 12, 24, and 48 h, cells were harvested and washed with PBS. These cells were examined for levels of ROS or NO generated and apoptosis, as mentioned below. For assessing levels of reactive oxygen species (ROS) in RAW264.7 cells, Cell ROX orange^TM^ (Thermo Fischer Scientific India Pvt Ltd, Mumbai, India) was added and cells were incubated for 30 min at 37°C. Stained cells were acquired in FACS Canto II cytometer (BD Biosciences) and data analyzed using FlowJo^TM^ software (Becton, Dickinson and Company, New Jersey, US).

The level of NO generated by macrophages was assessed using Griess reagent as per manufacturer's protocol. Briefly, 100 μl of Griess reagent [1% sulfanilamide in 2.5% H_3_PO_4_ and 0.1% naphtylethylenediamine in 2.5% H_3_PO_4_] was added to 100 μl of culture supernatants and absorbance was measured spectrophotometrically at 570 nm. Apoptosis was assessed using AnnexinV-7AAD staining kit (Biolegend, California, US) according to the manufacturer's instructions. Briefly, cells treated with Ms_Vc or Ms_Rv1507A were harvested, washed with cold PBS, and resuspended in binding buffer. Cells were stained with Annexin-7AAD stain, incubated for 15 min, and analyzed through flow cytometer FACS Canto II cytometer (BD Biosciences) using FlowJo^TM^ software. Cells treated with Staurosporine (500 nM) were used as positive control.

### Extracellular Staining of Surface Markers

RAW 264.7 cells or splenocytes (0.5 × 10^6^) from mice infected with Ms_Vc or Ms_Rv1507A were seeded in a 12 well plate, re-stimulated in absence or presence of recombinant Rv1507A protein (10 μg/ml) for 48 h. At the end of incubation, cells were washed with FACS buffer (PBS + 2% FBS) and treated with CD16/32. For surface staining, cells were treated with anti-mouse CD3, CD69, F4/80, CD19, CD4, CD8, CD86, CD80, MHCI, and MHCII antibodies and incubated on ice for 20 min. Cells were fixed using 4% paraformaldehyde. For assessing memory response, splenocytes from mice infected with Ms_Vc or Ms_Rv1507A were incubated in absence and presence of Rv1507A protein (10 μg/ml) for 96 h. Cells were washed, harvested, and stained with fluorescent antibodies against CD44 and CD62L memory markers. At least 20,000 live events were acquired through flow cytometer (BD LSRFortessa) and analyzed using FlowJo^TM^ software.

### Intracellular Staining for Cytokines

Splenocytes (1 × 10^6^) were seeded in 12-well plate and re-stimulated with Rv1507A (10 μg/ml) for 8 h in presence of Golgi plug^TM^ and Golgi stop^TM^ (BD Biosciences, San Diego, CA, USA). Cells were collected, washed with PBS, and stained with anti-CD4 and anti-CD8. Cells were fixed with 4% paraformaldehyde, permeablized in 0.02% triton X-100, followed by washing, and thereafter stained with anti-IFN-γ and anti-TNF-α for 1 h. Events were acquired by FACS and analyzed, as mentioned above.

### Mycobacterial Survival Assay

*Mycobacterium smegmatis* mc^2^155 grown till log phase was diluted at 1:100 in 7H9 media. Cells were cultured up to 12 h until OD_600_ reached 0.05. Re-inoculated cells were then allowed to grow for 30 h and the surviving cells were grown on 7H10 media after every 3 h in culture. OD_600_ was also taken after every 3 h up to 30 h.

RAW 264.7 cells were infected with Ms_Vc or Ms_Rv1507A at multiplicity of infection (MOI) of 1:10 for 4 h. The infected macrophages were washed 3 times and incubated in complete media supplemented with gentamicin. Cells were lysed in 1 ml of 0.025% SDS at various time points and aliquots of appropriate dilutions were plated on 7H11 agar plates. After 3 days of incubation, colonies were counted and survival rate was calculated as compared to the control. In a separate experiment, *M. smegmatis* mc^2^155 grown till log phase was diluted at 1:100 in 7H9 media. Cells were cultured up to 12 h until OD_600_ reached 0.2. Cells were re-inoculated in presence of H_2_O_2_ (5 and 10 mM) for 3, 6, or 9 h, and the surviving cells were grown on 7H10 media. In a separate experiment to test the susceptibility of strains to nitrosative stress, Ms_Rv1507A or Ms_Vc were incubated with 5 or 10 mM NaNO_2_ for 3 h, 6 h, or 9 h and surviving cells were grown on 7H10 media.

### Mycobacterial Phagocytosis Assay

Recombinant *M. smegmatis* (100 × 10^6^) Ms_Vc and Ms_Rv1507A were stained with SYTO-9 (10μM) (Thermo scientific) for 45 min. The stained cells were washed thrice with PBS to remove excess dye. RAW 264.7 cells were seeded in a 12 well-plate at (0.2 × 10^6^) and co-cultured with SYTO-9 stained Ms_Vc or Ms_Rv1507A at MOI 1:10 in incomplete DMEM for 4 h at 37°C. Cells were washed thrice with PBS and the SYTO-9 stained Ms_Vc or Ms_Rv1507A internalized by RAW 264.7 cells were analyzed through flow cytometer at various time points. For visualization of phagocytosis, RAW 264.7 cells were seeded on coverslips and allowed to adhere at 37°C in CO_2_ incubator. SYTO-9 stained cells were co-cultured with Ms_Vc or Ms_Rv1507A at MOI 1:10 in incomplete DMEM for 4 h at 37°C. The medium was aspirated, cells were fixed with PFA (2% in PBS) for 30 min. Excess PFA was quenched with 50 mM NH_4_Cl in PBS and visualized through fluorescence microscope (Nikon Carl Zeiss) (55).

### Histological Analysis of Lungs

BALB/c mice (*n* = 6) were injected with either Phosphate buffer saline (un-infected) or Ms_Vc (1 × 10^7^ CFU) or Ms_Rv1507A cells (1 × 10^7^ CFU) intra-tracheally. The lungs from representative mice were removed after 30 days of infection and fixed with 4% paraformaldehyde in phosphate-buffered saline (PBS). For histopathological examination the lungs were sliced into 1.5 × 1.0 cm slices with a surgical scalpel so that the thickness of the tissue was 5 mm. Each specimen was then labeled separately and then transferred to the automated tissue processor (Microm International GmbH, Germany). The tissue processor automatically processed the lung and spleen tissue to 12 cycles overnight which included 1 change in 10% formalin followed by graded dehydration in 2 changes each in 70% alcohol, 80% alcohol, 90% alcohol, respectively. The tissue was transferred to absolute alcohol to complete the dehydration. It was then subjected to 2 changes each in liquid chloroform for clearing followed by 2 changes with molten paraffin for embedding. The paraffin embedded tissues were then processed in TEC2800 cryoconsole for making blocks. The formalin-fixed, paraffin embedded blocks were sectioned into 4 μm ribbons using rotary microtome (Leica Biosystems Inc., USA). The sections were taken on floatation bath and placed over glass slides for staining and then rehydrated with 90% alcohol, 80% alcohol, 70% alcohol, respectively, and brought into distilled water. The sections were finally stained with hematoxylin and eosin for evaluation.

### Statistical Analysis

Statistical analysis was performed using GraphPad Prism8 software. Statistical significance was determined using ANOVA and Mann–Whitney test. *p* < 0.05 was considered significant, ^*^p < 0.05, ^**^p < 0.01, ^***^p < 0.001, and ^****^p < 0.0001. ROC curve analysis and the area under the curve (AUC) with 95% CI for Rv1507A was also calculated using GraphPad Prism. The optimal cut-off value was also determined from the ROC curve at maximal specificity and sensitivity to determine a value that could correctly classify patients and controls.

## Results

### *In-silico* Analysis and Knock-in of Rv1507A in *M. smegmatis* Reveals Its Mycobacterial Specificity and Immunogenic Potential

The hypothetical *M. tb* protein, Rv1507A, was examined to assess its role in modulating immune response in the host. The ORF of *M. tb* genomic sequence for possible coding segments that could translate into putative proteins was studied. A comparison of *M. tb* Rv1507A putative protein with known protein sequences available in database showed that it is absent in other species of mycobacteria including BCG (data not shown). *M. tb* Rv1507A also exhibited intrinsically disordered regions, but no protein binding sites, and possessed B cell epitopes (Figure S1) pointing to its antigenicity and immunogenicity. The secretory nature was predicted by PredictProtein software, which was further confirmed by western blot analysis of *M. tb* H37Rv culture filtrate (Figure S2). *M. tb* Rv1507A was cloned, expressed in pET28a vector, and the recombinant protein so obtained was purified (Figure S3). *M. tb* Rv1507A gene was also sub-cloned in pST-Ki expression vector and electroporated in non-pathogenic *M. smegmatis*. Positive knock-in constructs of *M. smegmatis* harboring His-tagged Rv1507A (Ms_Rv1507A) or Vector control pST-Ki (Ms_Vc) were cultured for further use (Figure S3).

### *M. tb* Rv1507A Activates Macrophages and Induces Pro-inflammatory Cytokine Response

**Figure 1:**
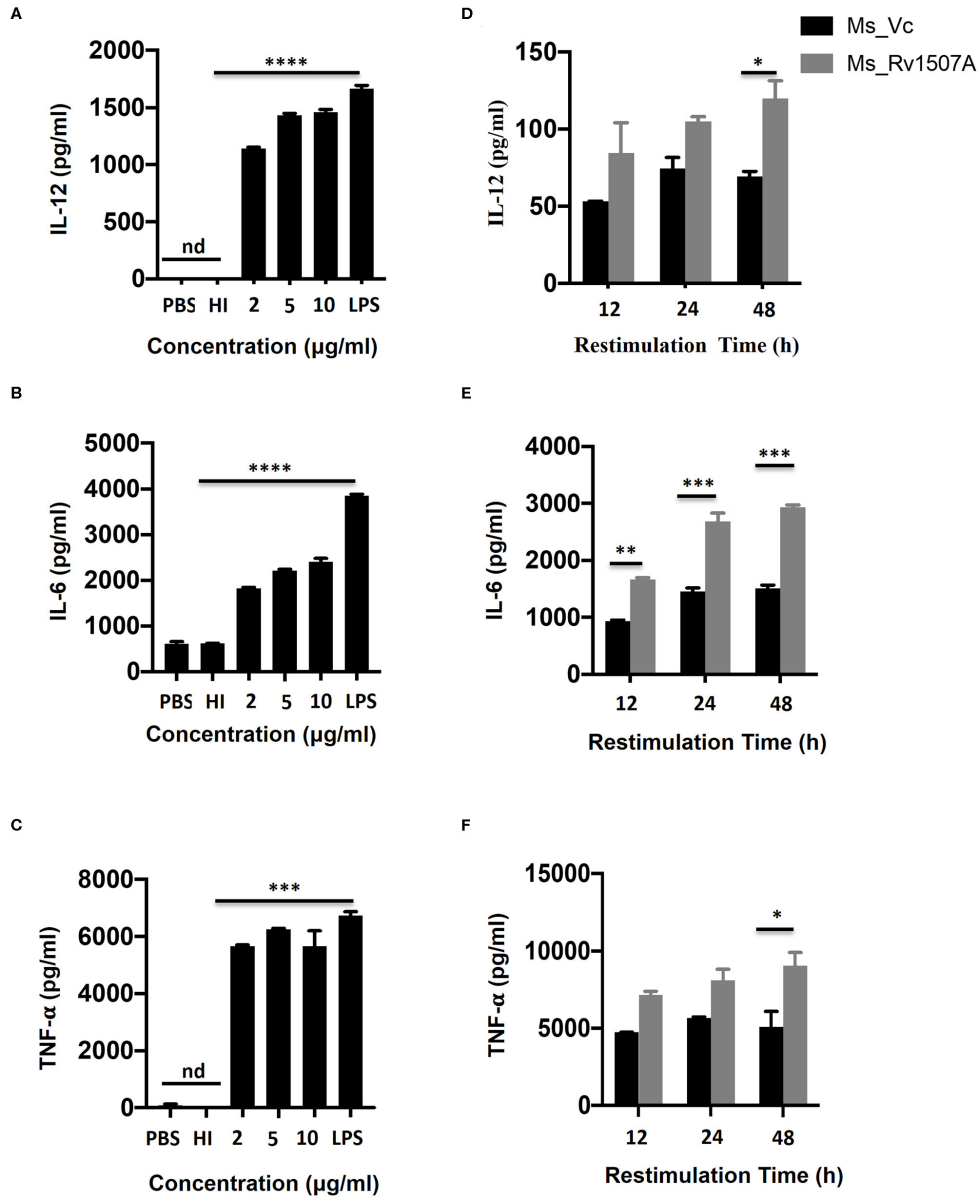
Rv1507A induces pro-inflammatory cytokines. RAW264.7 cells were treated with purified Rv1507A protein (2, 5, 10 μg/ml). LPS was used as positive control while PBS and heat inactivated (HI) protein served as negative controls. Levels of IL-12 **(A)**, IL-6 **(B)**, and TNF-α **(C)** were estimated using ELISA, as described in methods. Representative data from three experiments show the concentration of IL-12, IL-6, and TNF-α as mean ± SEM. Statistical significance was determined with one-way ANOVA. Additionally, spleen was recovered from BALB/c mice (*n* = 7) that were injected with Ms_Vc (1 × 10^7^) or Ms_Rv1507A (1 × 10^7^). Primed splenocytes were cultured *in-vitro* in presence of Rv1507A protein. Culture supernatants were harvested after 12 h, 24 h, and 48 h and concentrations of IL-12 **(D)**, IL-6 **(E)**, and TNF-α **(F)** were determined. Representative data from triplicate wells show the concentration of IL-12, IL-6, and TNF-α as mean ± SEM. Statistical significance was determined with two-way ANOVA. *P* < 0.05 was considered significant, *p < 0.05, **p < 0.01, ***p < 0.001, and ****p < 0.0001 (nd-not detected).

The role of *M. tb* putative protein Rv1507A in eliciting cytokine secretion was examined *in-vitro* by treating RAW264.7 macrophage cells with Rv1507A protein for 48 h. The Rv1507A protein, being recombinantly purified from *E.coli* BL21(DE3), could possibly contain LPS that may induce cytokine response and generate false results. In order to rule out this possibility, heat inactivated (HI) recombinant protein (Rv1507A) was used as control. The supernatant collected from RAW264.7 cells was assessed for levels of cytokines using ELISA. Rv1507A induced significant production of cytokines IL-12 (Figure 1A), IL-6 (Figure 1B), and TNF-α (Figure 1C) in macrophages. Moreover, RAW264.7 macrophage cells infected with Ms_Rv1507A also showed increased expression of CD86 (associated with maturation of APCs), CD40, and MHC I (associated with antigen presentation in APCs) (Figure S4). These results point to the ability of Rv1507A to enhance antigen presentation and co-stimulation for evoking an efficient immune response.

### *In-vivo* Exposure to *M. tb* Rv1507A Also Induces Pro-inflammatory Response and Up-Regulates Molecules Associated With Cell Maturation, Co-stimulation, and Antigen Presentation

**Figure 2:**
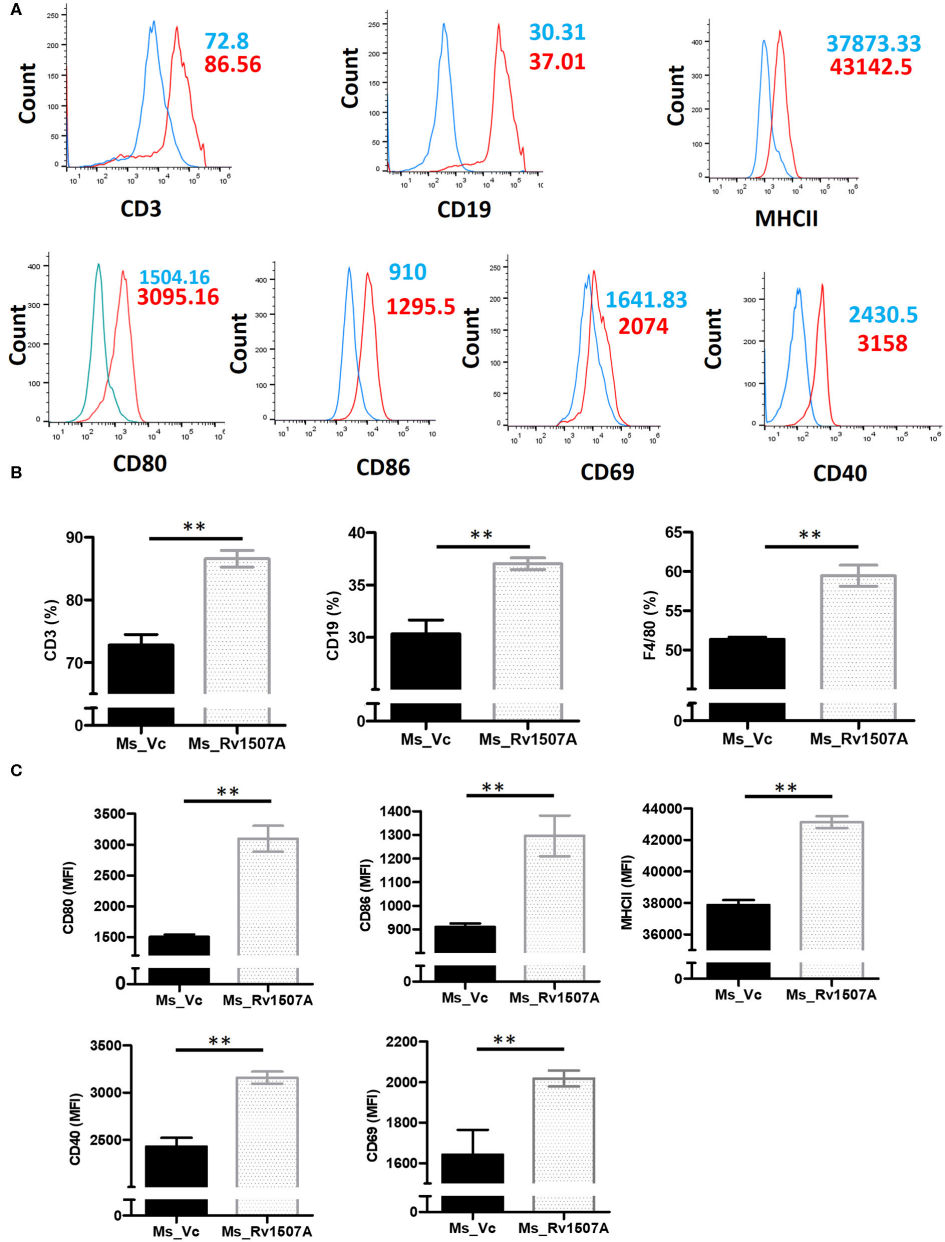
Rv1507A induces enhanced generation of lymphoid subpopulations. BALB/c mice (*n* = 6) were injected with Ms_Vc (1 × 10^7^) or Ms_Rv1507A (1 × 10^7^). Primed splenocytes obtained from mice were cultured *in-vitro* in presence of Rv1507A. The generation of CD3^+^ T cells, CD19^+^ B cells, and F4/80 macrophage cells, were measured by flow cytometry **(A,B)**. Note the increased generation of CD19^+^ B cells, CD3^+^ T cells, and F4/80^+^ macrophage cells. Expression of CD80, CD86, MHCII, CD40, and CD69 on primed splenocytes **(A,C)** are shown in MFI as mean ± SEM. *p* < 0.05 was considered significant, **p < 0.01.

The immunomodulatory role of Rv1507A was assessed by immunizing BALB/c mice with purified Rv1507A proteins and also by infecting with recombinant *M. smegmatis* (Ms_Rv1507A). Administration of Ms_Rv1507A resulted in splenomegaly (Figure S5A) in mice and increase in number of splenocytes (Figure S5B) as compared to Ms_Vc. Primed splenocytes, obtained from mice infected with Ms_Rv1507A or Ms_Vc, were re-stimulated with Rv1507A proteins (10 μg/ml). Culture supernatant from re-stimulated splenocytes was obtained after 12 h, 24 h, and 48 h. Re-stimulation of primed splenocytes with Rv1507A induces significantly higher IL-12 (Figure 1D), IL-6 (Figure 1E), and TNF-α (Figure 1F). These results point to the pro-inflammatory response elicited by Rv1507A protein. The effect of Ms_Rv1507A on generation of lymphocyte sub-populations was evaluated using multi-parameter flow cytometry. Results in Figures 2A,B show that Ms_Rv1507A induces significant (*p* < 0.01) increase in generation of CD3^+^ T cells, CD19^+^ B cells, and F4/80 macrophage cells. In order to ascertain the mechanistic basis of immunomodulation by Ms_Rv1507A, early activation marker (CD69), co-stimulatory receptors (CD80, CD86, and CD40), and antigen presenting molecules (MHC II) were examined. Results in Figure 2C show the mean fluorescence intensity (MFI), as an estimate of surface expression of these molecules on splenocytes. There is significant (*p* < 0.01) increase (Figures 2A,C) in expression of CD80, CD86, MHC II, CD40, and CD69 on specific cell populations in splenocytes from animals infected with Ms_Rv1507A as compared to Ms_Vc. It is evident that *M. tb* Rv1507A possibly modulates T cell activity through increase in expression of CD80 and CD86. Rv1507A can also modulate the activity of B cells through up regulation of CD40 which acts as co-stimulatory molecule and is required for association of APCs with T cells during antigen presentation. An increase in MHC II in presence of Ms_Rv1507A points to their possible role in modulating antigen presentation to CD4^+^ cells.

### Rv1507A Exposure Generates Polyfunctional T Cells and Cells Having Memory Like Phenotype

**Figure 3:**
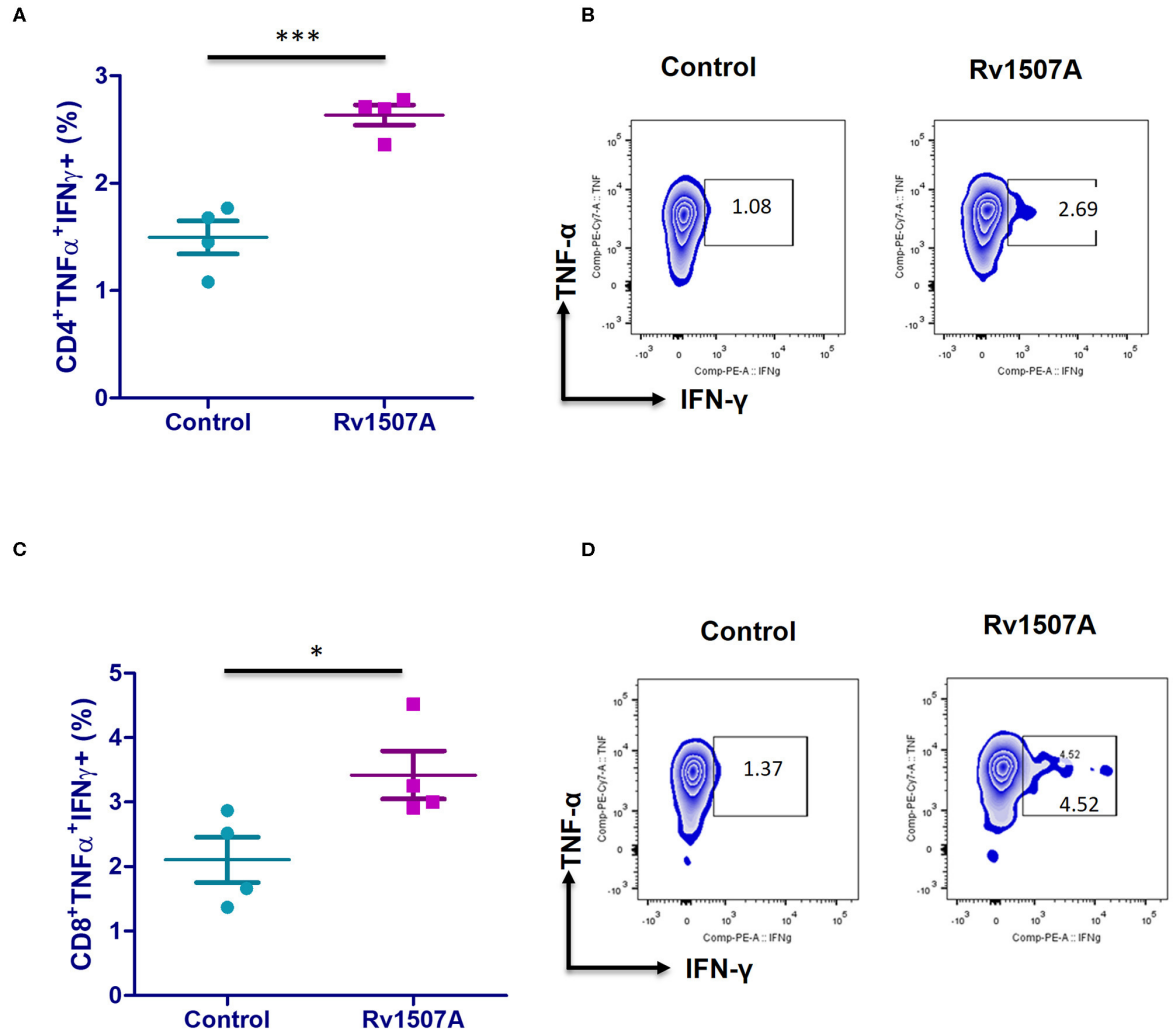
Rv1507A induces generation of poly-functional IFN-γ and TNF-α secreting CD4^+^ and CD8^+^ cells. BALB/c mice were immunized with either purified Rv1507A proteins (10 μg/ml) or PBS alone. Splenocytes obtained from mice were cultured in absence or presence of Rv1507A protein (10 μg/ml) for 48 h and the intracellular levels of IFN-γ and TNF-α were estimated, as described in methods. The percentage of CD4^+^ TNF-α^+^IFN-γ^+^ cells **(A)** and CD8^+^ TNF-α^+^IFN-γ^+^ cells **(C)** are shown as mean ± SEM. Representative corresponding density plots of CD4^+^ TNF-α^+^IFN- γ^+^ cells **(B)** and CD8^+^ TNF-α^+^IFN-γ^+^ cells **(D)** are shown. Statistical significance was determined with one tailed Mann–Whitney test. *p* < 0.05 was considered significant, *p < 0.05, ***p < 0.001.

**Figure 4:**
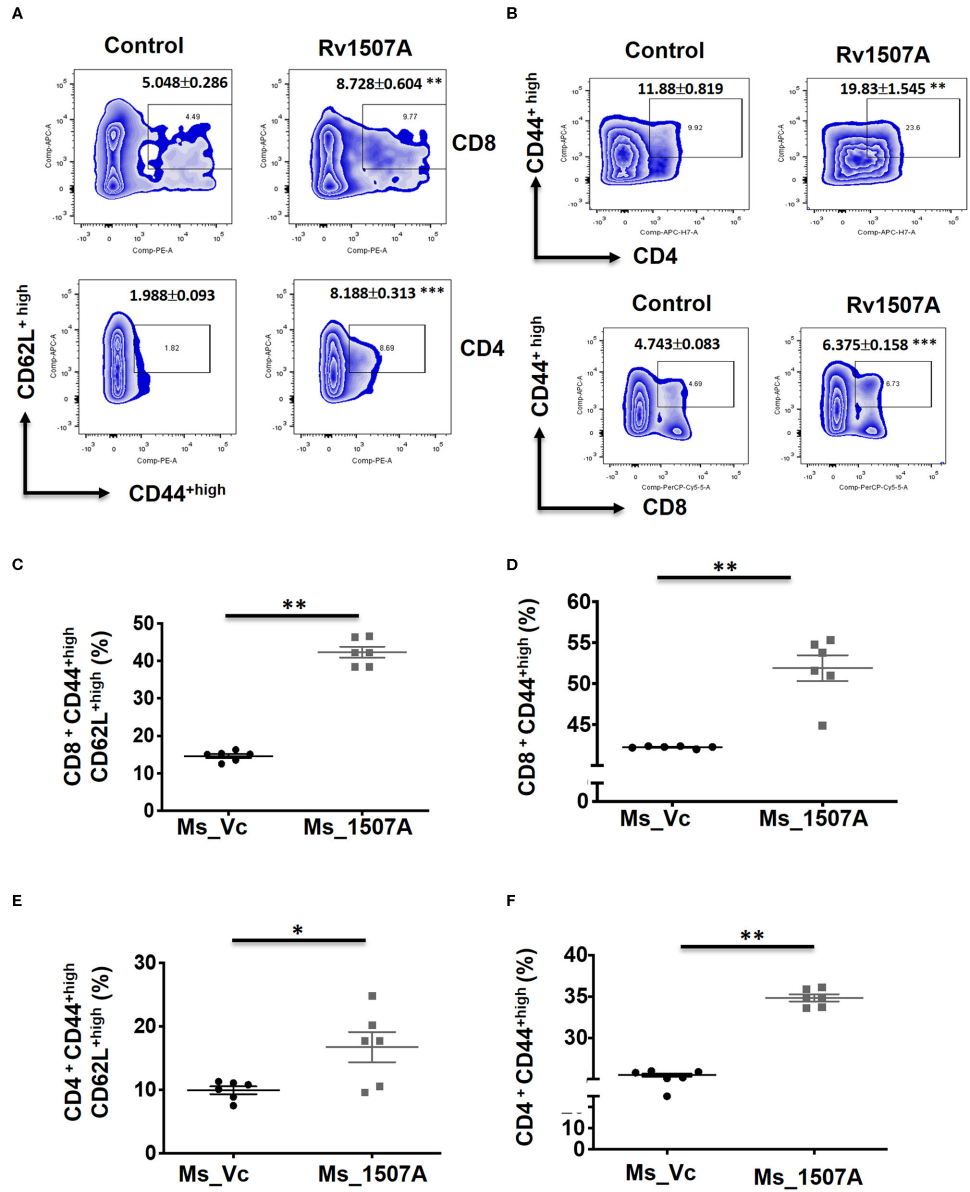
Rv1507A protein and recombinant Ms_Rv1507A mount effector and central memory like response. Spleens were recovered from BALB/c mice that were either injected with purified Rv1507A proteins (10 μg/ml) or PBS alone. Splenocytes were cultured in presence of Rv1507A, *in-vitro* for 96 h. Representative density plots of CD8^+^CD44^high^CD62L^high^ (T_CM_) cells and CD4^+^CD44^high^CD62L^high^ cells are shown **(A)**. CD8^+^CD44^high^ (T_EM_) cells and CD4^+^CD44^high^ cells are shown in **(B)**. Additionally, splenocytes from mice infected with Ms_Vc (1 × 10^7^) or Ms_Rv1507A (1 × 10^7^) were re-stimulated with Rv1507A *in-vitro* for 96 h. Percent of CD8^+^ cells expressing CD44^high^CD62L^high^ **(C)** and CD44^high^ cells **(D)** were measured by flow cytometry, shown as mean ± SEM. Similarly CD4^+^ T_CM_ and T_EM_ were quantified and depicted in **(E,F)**, respectively. Statistical significance was determined with one tailed Mann–Whitney test. *p* < 0.05 was considered significant, *p < 0.05, **p < 0.01.

The above results presented so far pointed to the modulation of T cell responses by Rv1507A. The role of Rv1507A in generating polyfunctional T cells was studied by infecting mice with Ms_Rv1507A or Ms_Vc or else by immunizing with recombinant purified Rv1507A protein. The primed splenocytes were obtained after 4 weeks and re-stimulated with Rv1507A protein. In response to *in-vitro* protein stimulation, a significant (*p* < 0.001) increase in number of double-positive (IFN-γ^+^/TNF-α^+^) CD4^+^ cells (Figures 3A,B) as well as CD8^+^ cells (*p* < 0.05) (Figures 3C,D) was observed in immunized mice. These observations demonstrate that Rv1507A induces antigen-specific multifunctional CD4^+^/CD8^+^ T cells in mice, thereby pointing to its role in modulating T cells function in response to infections. This observation was validated by estimation of IFN-γ from culture supernatants, which revealed enhanced secretion of this classical Th1 cytokine from Rv1507A immunized animals (Figure S6). Sub-populations of CD4^+^ or CD8^+^ cells expressing CD44 and CD62L were examined by flow cytometry. Rv1507A induced significant (*p* < 0.05) memory like response with increase in central memory response (CD8^+^CD44^+^CD62L^+^ T cells, CD4^+^CD44^+^CD62L^+^ T cells) and effector memory (CD4^+^CD44^+^ T cells, CD8^+^CD44^+^ T cells) as shown in Figures 4A,B, respectively. Consistent with the above results, Ms_Rv1507A also induced strong central (Figures 4C,E) and effector memory response (Figures 4D,F).

### *M. tb* Rv1507A Induces IgG Response That Exhibits Sero-Specificity

**Figure 5:**
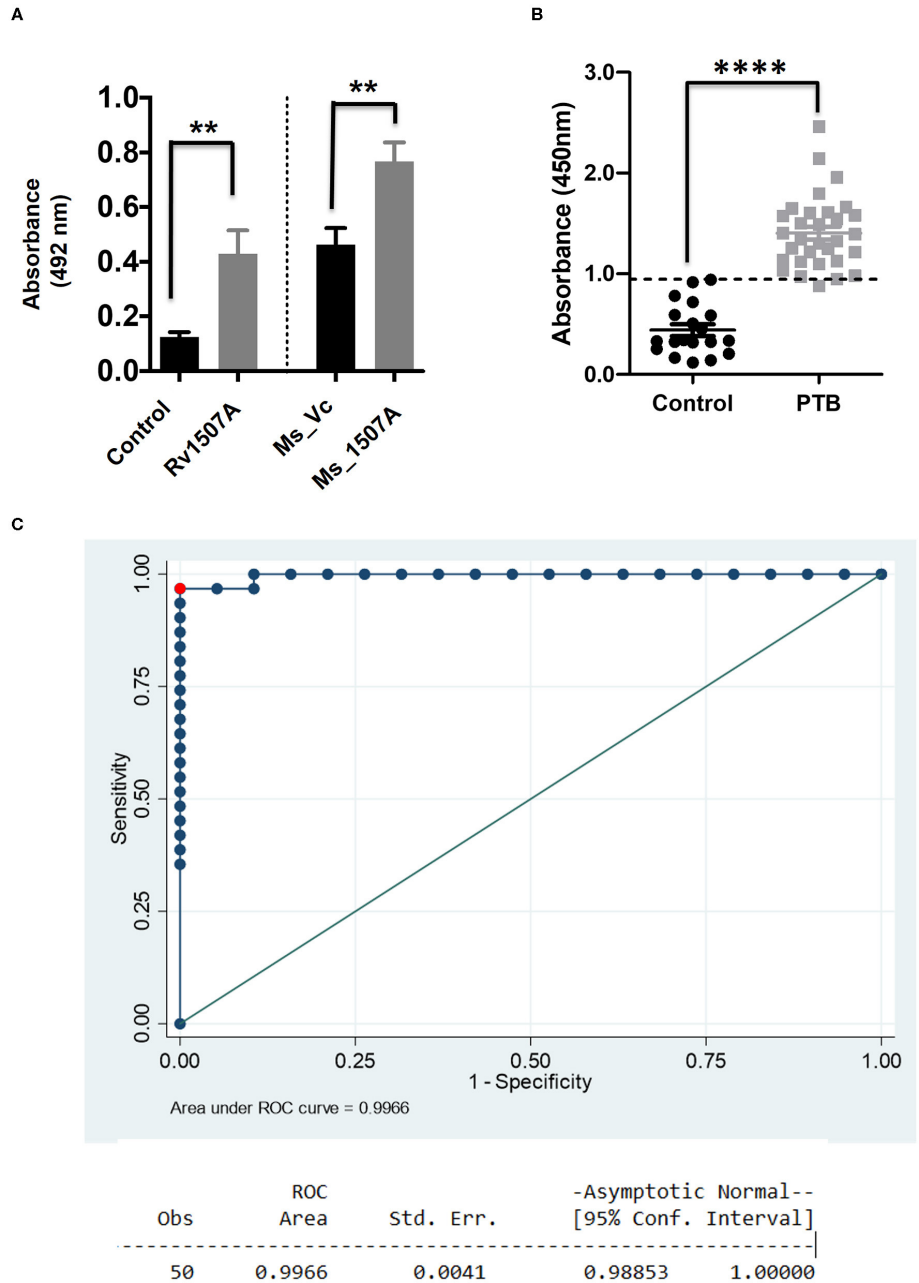
*M. tb* Rv1507A protein elicits IgG response in immunized mice and exhibits immunoreactivity in sera of Tuberculosis patient. BALB/c mice were immunized with either purified *M. tb* Rv1507A protein or infected with Rv1507A knock-in *M. smegmatis* (Ms_Rv1507A) and control *M. smegmatis* (Ms_Vc). The level of IgG in the mouse sera was tested using ELISA and measured spectrophotometrically (492nm) **(A)**. Sera were collected from pulmonary TB patients (*n* = 31) or control volunteers (*n* = 19). Immunoreactivity of human sera against Rv1507A was tested as described in methods and measured spectrophotometrically (450 nm) **(B)**. Receiver operating characteristics (ROC) curve to assess the cut-off value of ELISA depicting % sensitivity and % specificity **(C)**. Data are shown as mean ± SEM. Statistical significance was determined with one tailed Mann–Whitney test. *p* < 0.05 was considered significant. **p < 0.01, ****p < 0.0001.

Having shown the ability of Rv1507A to generate polyfunctional T cells as well as memory cells, we further assessed its effect on generation of humoral immune response. The immunoglobulin G (IgG) response from the sera of mice immunized with purified Rv1507A protein or Rv1507A knock-in *M. smegmatis* was done using ELISA. Mice treated with Rv1507A protein elicited nearly two-fold higher titer of IgG as compared to control mice. Similarly, mice injected with Ms_Rv1507A elicited nearly two-fold higher IgG response as compared to mice injected with Ms_Vc (Figure 5A). These results point to the immunogenecity of Rv1507A in eliciting IgG response. Following this observation, humoral immune responses directed against the Rv1507A protein were also compared in pulmonary tuberculosis (PTB) patients and healthy controls. Sera of all the PTB patients showed significantly (*p* < 0.001) higher IgG reactivity against Rv1507A protein antigen as compared to sera of the healthy controls (Figure 5B). Based on these OD values, an ROC analysis was performed that revealed optimal cut-off for the assay to distinguish patients and controls. At cut-off OD values of 0.947, sensitivity of 96.7% with 100% specificity was achieved. Area under Curve (AUC) value being 0.996 (95% CI, 0.988–1.000) revealed an excellent diagnostic efficacy of Rv1507A (Figure 5C). These results point to the specificity of Rv1507A, and also its value as a novel diagnostic marker.

### Rv1507A Knock-in *M. smegmatis* Shows Increased Uptake in Macrophages and Associated Stress Responses

**Figure 6:**
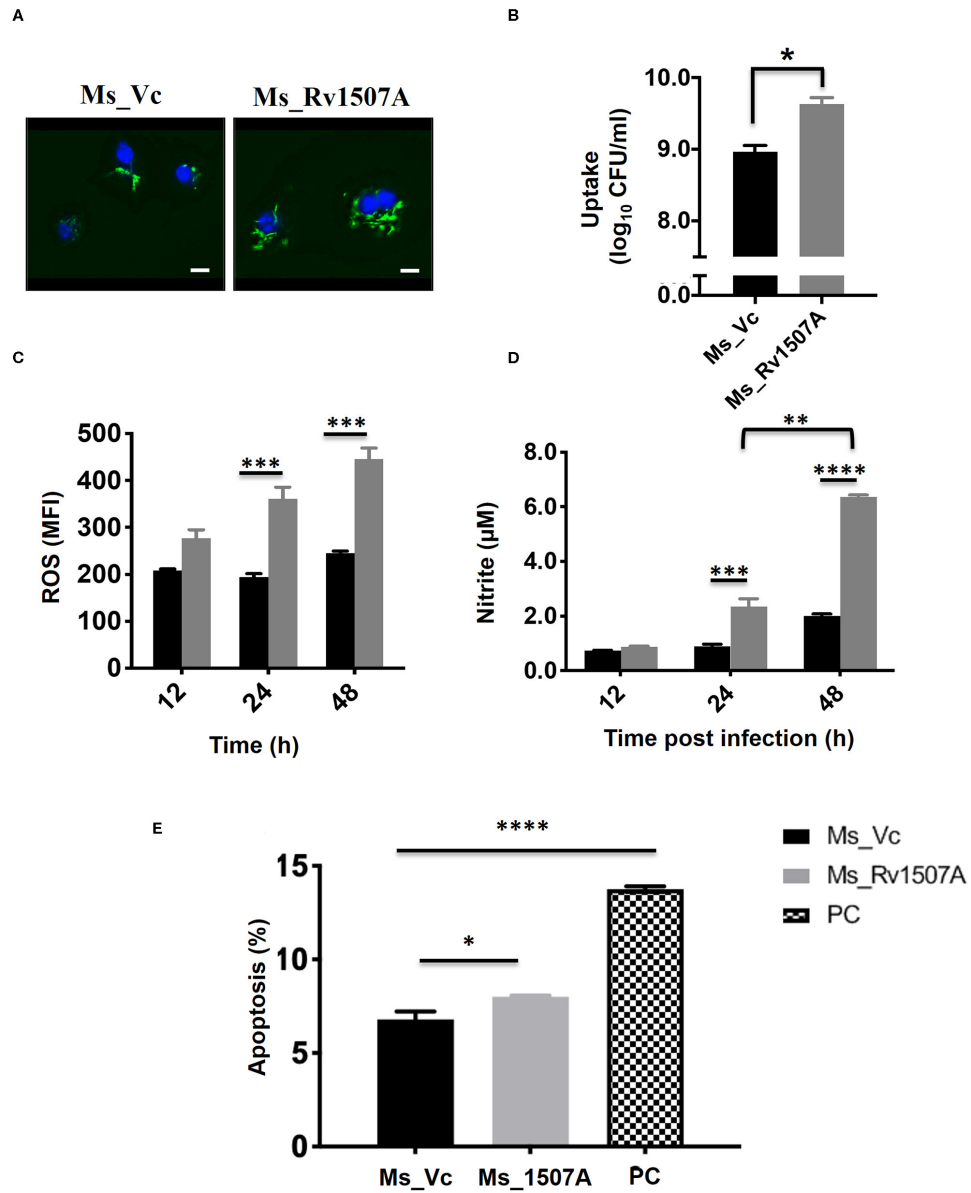
Rv1507A knock-in *M. smegmatis* exhibits enhanced uptake by macrophage and induction of stress response. RAW 264.7 macrophage cells infected with fluorescent SYTO-9 stained Ms_Vc and Ms_Rv1507A. The uptake of Ms_Vc and Ms_Rv1507A within the cells were visualized microscopically (magnification 100X, scale bar represents 5 μm) **(A)** and by CFU assay **(B)**. RAW 264.7 cells infected with Ms_Vc (black box) and Ms_1507A (gray box) were stained with Cell ROX Orange and assessed for reactive oxygen species (ROS). Representative data shows the mean fluorescence intensity (MFI) of ROS generated within the macrophage as mean ± SEM from three separate experiments **(C)**. Statistical significance was determined by two-way ANOVA. Cell culture supernatants were assessed for levels of Nitric Oxide (NO), using Griess reagent assay, after 12, 24, and 48 h post infection of RAW264.7 cells with Ms_Rv1507A (gray box) or Ms_Vc (black box). Representative data show the concentration of NO as mean ± SEM from three separate experiments **(D)**. Statistical significance was determined by two-way ANOVA. RAW264.7 cells were cultured in presence of Ms_Vc or Ms_Rv1507A at an MOI of 10:1. After 48 h, cells were stained with AnnexinV-7AAD dye and apoptosis was assessed. Representative data show the percent apoptotic cells as mean ± SEM from three separate experiments. Cells treated with Staurosporin (500 nM) were used as a positive control (PC) **(E)**. Statistical significance was determined by one-way ANOVA. *p* < 0.05 was considered significant, *p < 0.05, **p < 0.01, ***p < 0.001, and ****p < 0.0001.

Sustained expression of Rv1507A in recombinant *M. smegmatis*, confirmed by western blot, revealed successful integration (Figure S7A). A comparison of growth kinetics of Ms_Rv1507A and Ms_Vc culture showed no significant difference in doubling time (Figure S7B). Fluorescence microscopy of SYTO-9 stained bacteria showed that increased number of Ms_Rv1507A were localized in the cytoplasm of macrophage cells as compared to Ms_Vc (Figure 6A). The infectivity of recombinant Ms_Rv1507A was assessed by their ability to infect cells in a given time as determined by CFU. Higher CFU load suggested that non-pathogenic *M. smegmatis* acquired infectivity by virtue of incorporation and expression of Rv1507A in recombinant *M. smegmatis* (Figure 6B).

The physiological responses in RAW264.7 cells due to infection with Ms_Rv1507A or Ms_Vc were assessed every 12 h by estimating ROS or NO generated and induction of apoptosis. Results in Figure 6C showed that RAW264.7 cells infected with Ms_Rv1507A generated nearly two-fold higher (*p* < 0.001) ROS at 24 and 48 h as compared to infection with Ms_Vc. Results in Figure 6D show that Ms_Rv1507A induces generation of NO, nearly two-fold higher (*p* < 0.001) at 24 h and three-fold higher (*p* < 0.0001) at 48 h, as compared to cells infected with Ms_Vc. These results show that upon infection with Rv1507A knock-in *M. smegmatis*, RAW264.7 cells exhibit stress response through generation of ROS and NO. RAW264.7 cells infected with Ms_Rv1507A also showed reduced viability as compared to cells infected with Ms_Vc. In order to assess the mechanistic basis of reduction in viability of RAW264.7 cells due to infection with Ms_Rv1507A, cells were stained with AnnexinV-7AAD and percent apoptotic cells were assessed by flow cytometry. Results in Figure 6E show that Ms_Rv1507A induced significant (*p* < 0.05) increase in apoptosis of RAW264.7 cells at 48 h post infection as compared to Ms_Vc. The percent apoptotic RAW264.7 cells were not significant at 12 or 24 h (data not shown). It is evident that infection with Ms_Rv1507A resulted in stress response in RAW264.7 cells, leading to apoptosis.

### Increased Survivability of Rv1507A Knock-in *M. smegmatis* Within Macrophage and Other Stress Conditions

**Figure 7:**
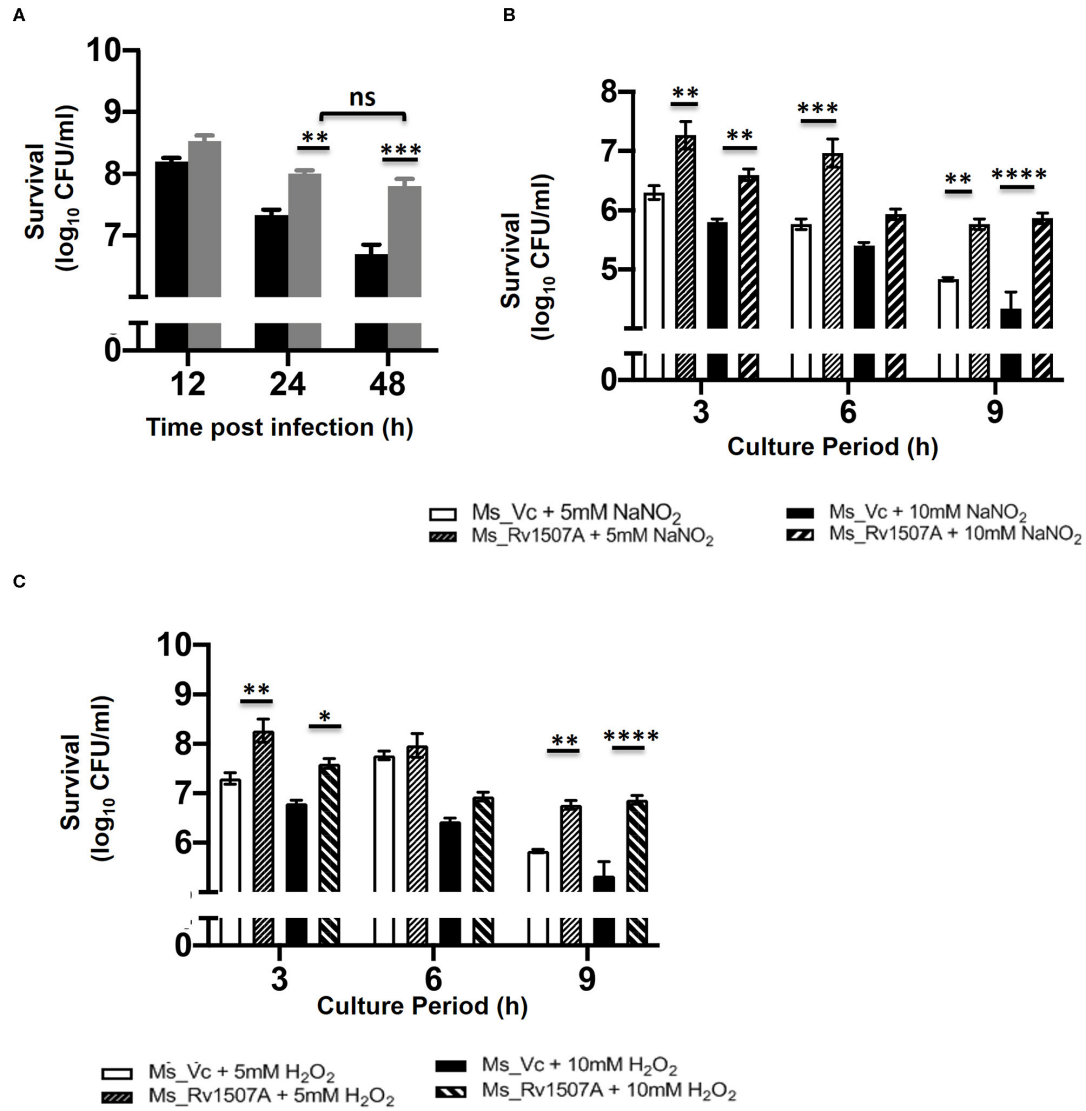
Increased survivability of Ms_Rv1507A within macrophages and resistance to stress induction in culture. RAW 264.7 cells were co-cultured with Ms_Vc or Ms_Rv1507A and the colony forming units/ml were counted after 12 h, 24 h, and 48 h. Representative data from three experiments show survival of viable Ms_Vc (black box) and Ms_Rv1507A (gray box) as mean±SEM **(A)**. Ms_Vc and Ms_Rv1507A cells were cultured in presence of 5 and 10 mM of sodium nitrite. The cell viability was assessed at 3 h, 6 h, and 9 h. Representative data from three experiments show the survival in terms of log_10_ CFU/ml as mean±SEM **(B)**. Ms_Vc and Ms_Rv1507A cells were cultured in presence of 5 and 10 mM of hydrogen peroxide. The cell viability was assessed at 3, 6, and 9 h. Representative data from three experiments show the survival in terms of log_10_ CFU/ml as mean ± SEM **(C)**. Statistical significance was determined by two-way ANOVA. *p* < 0.05 was considered significant, *p < 0.05, **p < 0.01, ***p < 0.001, and ****p < 0.0001.

The survivability of Ms_Rv1507A within macrophages at different time points, post-infection, was examined by enumerating CFU. The CFU/ml of Ms_Rv1507A or Ms_Vc corresponds to the number of bacteria that survived within the macrophage before being plated on agar plates. Ms_1507A exhibits significantly (*p* < 0.01) higher survivability within macrophages for longer durations (Figure 7A). The survivability of Ms_Vc decreased at 24 and 48 h of infection compared to Ms_Rv1507A. These results demonstrate that Ms_Rv1507A possess increased survivability within the macrophage. Similarly, RAW264.7 cells infected with Ms_Rv1507A showed about two-fold higher MFI that corresponds to increased uptake of SYTO-9 stained bacteria as compared to Ms_Vc (Figure S8).

Enhanced levels of ROS and NO in macrophages can effectively get rid of *M. smegmatis* infection (56). However, our results show that Ms_Rv1507A survives for prolonged duration within the macrophages (Figure 7A). Ms_Vc or Ms_Rv1507A were treated with sodium nitrite (5 and 10 mM) and hydrogen peroxide (5 and 10 mM) for up to 9 h to induce NO and ROS stress, respectively. The CFU of Ms_Vc or Ms_Rv1507A as a measure of viable bacteria that survived in the presence of sodium nitrite and hydrogen peroxide were examined. A significantly higher CFU of Ms_Rv1507A as compared to Ms_Vc was observed after treatment of respective cultures with 5 mM sodium nitrite for 3 and 9 h or with 10 mM sodium nitrite for 3, 6, and 9 h (Figure 7B). Similarly, cultures treated with hydrogen peroxide (5, 10 mM) for 3 and 9 h showed significantly higher CFU of Ms_Rv1507A cells as compared Ms_Vc cells (Figure 7C). These results showed that Ms_Rv1507A is more resilient to NO or ROS stress as compared to Ms_Vc. These results provide a logical interpretation of our earlier results that showed that Ms_Rv1507A survived oxidative stress within macrophages. Therefore, Rv1507A protein aids in survival of mycobacteria under stress conditions generated within immune cells in response to infections.

### Intra-tracheal Infection of Ms_Rv1507A Reveals Increased Infiltration of Lymphocytes in the Lungs

**Figure 8:**
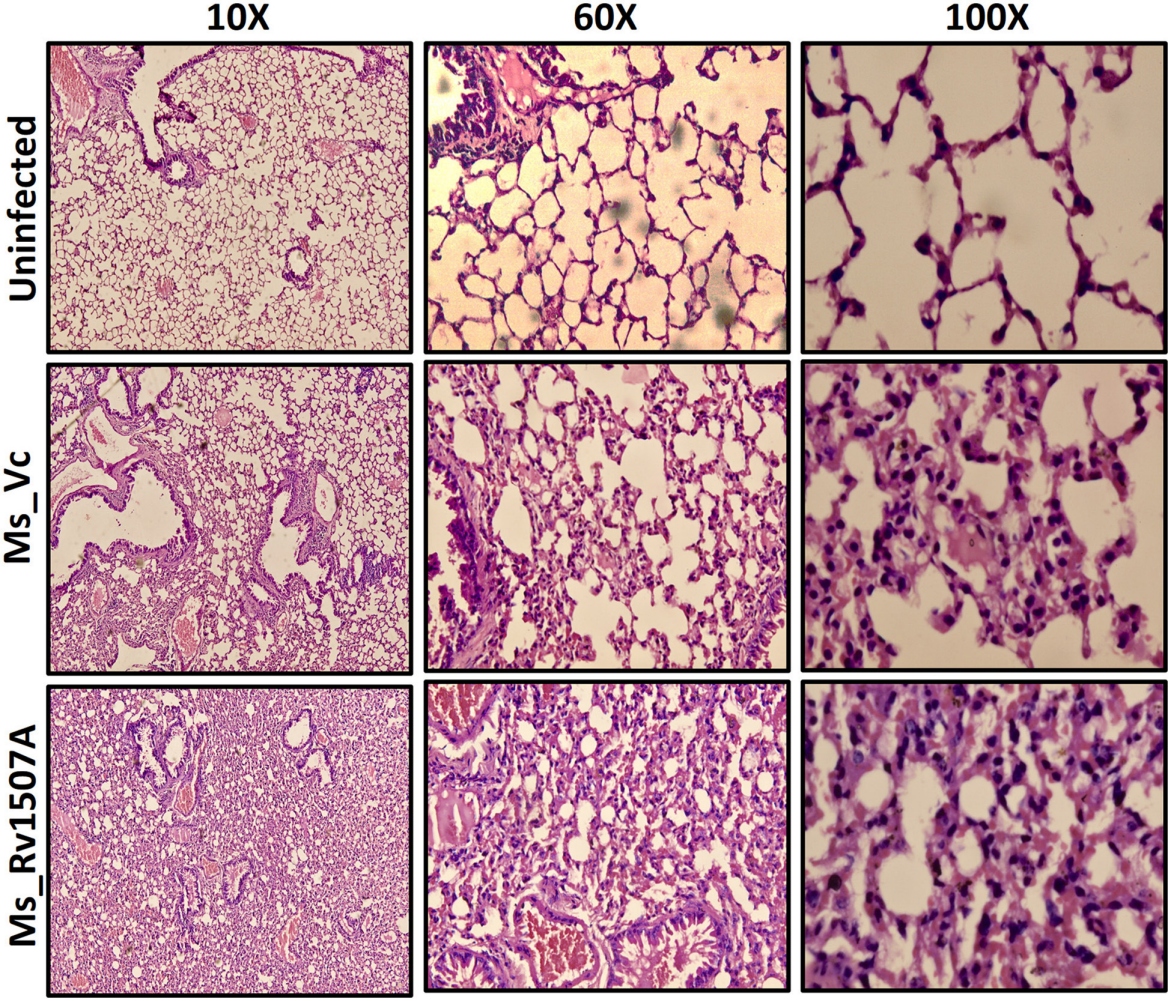
Ms_Rv1507A increases the infiltration of lymphocytes in the lung tissue of immunized mice. Lungs were recovered after 30 days from BALB/c mice (*n* = 6) that were injected with either Phosphate buffer saline (un-infected) or Ms_Vc (1 × 10^7^) or Ms_Rv1507A cells (1 × 10^7^) intratracheally. Lungs were washed in PBS and then fixed in 10% formalin solution. After fixation, lungs were fine–sectioned and stained with Hematoxylin and Eosin (HE) solution. Eosin is pink and stains proteins non-specifically. In a typical tissue, nuclei are stained blue, whereas the cytoplasm and extracellular matrix have varying degrees of pink staining. The images were captured for at least 5 different fields in 6 mice.

After exploring the immunological response of Rv1507A and the increased persistence of Ms_Rv1507A in macrophages along with stress response related correlates, we assessed its effect via aerogenic route to mimic actual infection. Histological analysis of lung tissue after aerogenic infection revealed enhanced infiltration of lymphocytes into lung parenchyma (Figure 8) in animals infected with Ms_Rv1507A as compared to vector control (Ms_Vc). There was no evidence of any granuloma like structure or any caseous necrotic lesions after intra-tracheal instillation. These observations suggest that Ms_Rv1507A was more immunogenic as compared to Ms_Vc but was not virulent enough to cause any adverse pathology. All these observations point to the possibility of Rv1507A as a likely vaccine candidate. This however, needs to be evaluated further. More importantly the comparison with BCG in terms of providing protection against *M. tb* infection needs to be considered to validate its vaccine efficacy.

## Discussion

Given the goal to eradicate TB by 2035 (3), it is imperative to embark on parallel strategies based on new drugs, repurposed drugs, and novel vaccines with emphasis on route of administration to generate protective immunity (7, 57, 58). In this study, we evaluated the pro-host and possible vaccine potential of antigenic protein Rv1507A, which is absent in BCG strain. In depth *in-silico* analysis of Rv1507A showed that the protein is enriched with alpha helix and beta sheets with a disordered domain in C-terminal region. These disordered regions provide structural plasticity and conformational adaptability to proteins to enhance their binding promiscuity with various ligands. This could help pathogens to compensate for the genome reduction due to reductive evolution (59) by resorting to disordered proteins, moonlighting functions and protein promiscuity (45, 60, 61). *In-silico* analysis for epitope prediction and antigenicity revealed that this protein is highly antigenic. Rv1507A, being unique for *M. tb* and especially absent in BCG (Data in communication), could also be a potential immunodiagnostic biomarker for tuberculosis. This specificity toward pathogens reveals that these pathogen specific proteins must be necessary for survival inside host and might have been lost through reductive evolution by non-pathogens (59). These proteins are thus at the forefront of host pathogen interaction and need to be explored for their possible use as diagnostics or as vaccine candidates.

**Figure 9:**
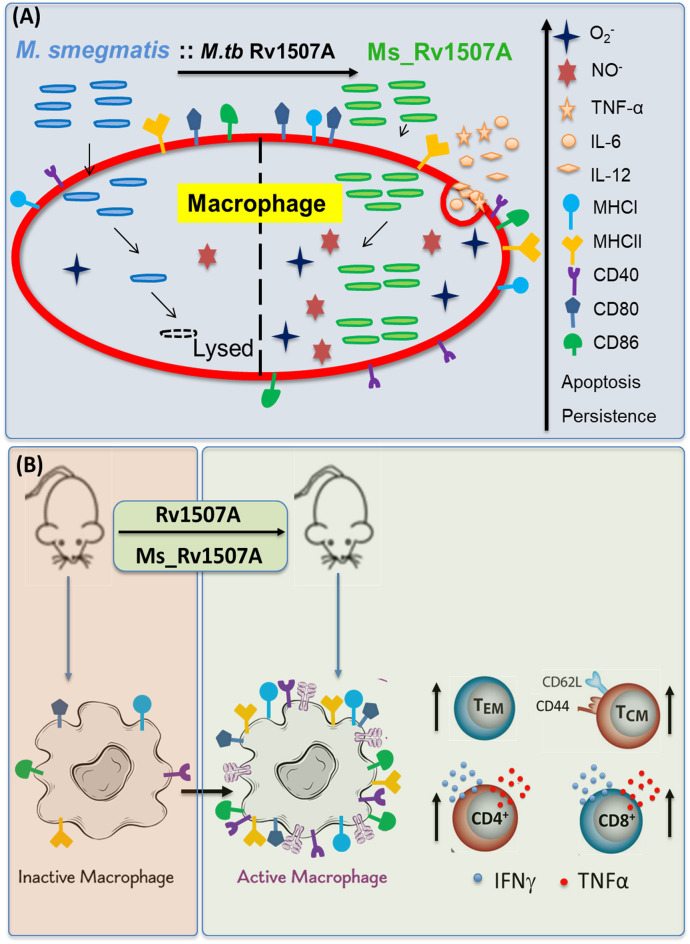
Model depicting the immunomodulatory role of Rv1507A. **(A)** Recombinant *M. smegmatis* expressing Rv1507A is phagocytosed by macrophage which increases generation of ROS and NO that leads to induction of apoptosis. Macrophage exposed to Rv1507A secretes pro-inflammatory cytokines such as TNF-α, IL-12, and IL-6. It also leads to increase in the expression of MHCI, MHCII, CD40, CD80, and CD86 that indicates efficient antigen presentation and co-stimulation. **(B)** Immunization with Rv1507A protein or Ms_Rv1507A in mice leads to increased humoral and cell mediated immunity. A robust effector memory (T_EM_) and central memory (T_CM_) response is also evoked along with enhanced poly-functional CD4^+^ as well as CD8^+^ cells.

Macrophages, being the sentinels of the immune system, are the primary cells that encounter pathogen. *M. tb* has developed different strategies to invade and adapt inside macrophages and dendritic cells that form the primary defense against infections (62). Macrophages and dendritic cells are antigen-presenting cells that modulate function of other cells through cytokines. Cell-mediated immunity governed by cytokines such as IFN-γ and TNF-α are mainly responsible to inhibit the growth of *M. tb* and clear the infection (63). Previous studies on mice which were knocked down for pro-inflammatory genes like IFN-γ and TNF-α showed these to be more prone to *M. tb* infection (64–66). The pro-inflammatory cytokine milieu generates Th1 protective response that allows efficient clearance of bacteria. The role of IL-6 cytokine is controversial as it has been found to have both protective and pathological roles in TB infection. During early phase of infection, it is involved in protection; while in chronic phase of TB infection, it is associated with pathology (67). Evaluation of IL-6 levels from the collected supernatant from RAW264.7 cells exhibited dose dependent increase. Treatment of Rv1507A also activates innate immunity by activating macrophages that is evident by enhanced expression of co-stimulatory markers on these professional APCs. CD80 and CD86 provide co-stimulatory signal to T cells *via* CD28 during antigen presentation and are responsible for effective activation of T cells for proliferation and cytokine production (68, 69). CD40 is another co-stimulatory molecule that activates APCs upon interaction with CD154 (CD40L) on CD4 T cells (70). This activation translates into effective microbicidal response by these APCs through NO and ROS that also modulate initiation and progression of cellular and humoral adaptive immunity. CD69 is an early activation marker of T cells and can be induced on other cells of hematopoietic lineage. Enhanced expression of these molecules suggests presence of activated T cells that indicates effective T cell immunogenicity that corroborates *in-silico* data for T cell epitope prediction. This activation of innate receptors generates antimicrobial response apart from effective activation of adaptive arm of immunity (Figure 9).

Adaptive immune cells like CD4^+^ and CD8^+^ T cells modulate the function of other immune cells that in turn maintain checks and balances on *M. tb* infection (71, 72). CD4^+^ and CD8^+^ T cells are important during acute phase of infection and in clearance of chronic infection, respectively (73, 74). Moreover, cytotoxic CD8^+^ T cells play an important role, through MHC I pathway, in protection against intracellular pathogens. CD4^+^ T cells on the other hand play a crucial role in response to antigen processed by APCs through MHC II pathway. These cells differentiate into different effector subtypes and modulate the cytokine profile which leads to cellular proliferation of B cells and activation of antigen presenting cells (APCs). The classical approach to estimate the vaccine efficacy of a candidate protein is to evoke Th1 response that is usually considered protective in case of intracellular infections. The hallmark Th1 cytokine IFN-γ activates macrophages for pathogen clearance and TNF-α acts in synergy to produce NO to unleash bacteriostatic effect of macrophages. TNF-α also modulates migratory potential and thus leads to granuloma formation to ward off the bacteria from further transmission (23). We observed induction of pro-inflammatory cytokine (IFN-γ) in immunized mice, which clearly depicts pro-host immune modulatory effect of the antigenic protein (Figure S5). This immune effect was devoid of any bias as we avoided use of any adjuvant due to their inherent ability to skew resultant immune response toward Th1 or Th2 depending on their physiochemical properties (47, 48, 75, 76). The adjuvants are required to activate innate immune receptors, such as pattern recognition receptors (PRRs), toll-like receptors (TLRs), nucleotide-binding oligomerisation domain (NOD)-like receptors, or retinoic acid-inducible gene-I (RIG-I)-like receptors, each of which initiate different downstream cytokine signaling to mediate protection against pathogens. Treatment of macrophages with mycobacterial proteins reveals their self-adjuvant properties as evident from up-regulation of various co-stimulatory receptors. Adjuvant role of mycobacterial proteins has been earlier delineated and our observations also reflect the same (77, 78). The secretion of TNF-α and IFN-γ were found to be significant from CD4^+^ as well as CD8^+^ T cells of Rv1507A sensitized mice as compared to control. Even though our observations also suggest polyclonal activation by antigenic stimulation, as revealed by most cells showing TNFα secretion, there was a distinct antigen induced increase in cells secreting both TNFα and IFNγ upon immunization. These poly-functional T cells are one of the classical predictors of protective immunity and act as a signature of vaccine induced protection (79). It is conceivable that cells expressing multiple effector molecules would be more effective to control pathogens as these cytokines act synergistically. Various other *M. tb* antigens, which form the components of vaccines in clinical trials, have been shown to elicit poly-functional CD4^+^ T cells (79). The other functional attributes of these polyfunctional cells include their enhanced potency for cytokine secretion and long-lived memory function with ability to migrate into lungs. We also analyzed the memory like phenotype of T cells after Rv1507A immunization as well as after infection with Ms_Rv1507A. T_EM_ are involved in immediate responses and induce cytolysis of infected cells. These cells are devoid of homing receptors thus are usually present in blood or peripheral tissues. T_CM_ on other hand are highly proliferative with ability to generate effector cells. These cells have longer life span and are involved in long term protection (80).

Apart from cell mediated immunity, Rv1507A also evoked humoral immunity in mice as revealed by increased levels of protein specific antibodies in immunized animals. Although the role of antibodies in protection against TB has been subjugated by cell mediated immunity however recent evidence suggests that antibodies can positively modulate the immune responses against *M. tb*. It is evident that multifaceted B cells, by virtue of antigen presentation, antibody and cytokine production, are able to exert significant effect on T cell mediated immunity that is classically considered as critical for TB control (81–83). Extrapolating these findings to human host we expected antibodies against Rv1507A in sera of TB patients. A clear non-overlap in IgG reactivity in PTB patients and healthy controls points to the fact that response by Rv1507A is highly specific and attests our observation that Rv1507A is absent in mycobacteria except *M. tb*. The credibility of a diagnostic test is usually measured by AUC of an ROC curve. The AUC value of 0.996 depicts excellent diagnostic utility of this protein. Likelihood ratio (LR) of a test is also an important statistical method to better evaluate the diagnostic test. In our study, LR of positive test values was very high with lower corresponding LR negative values (Figure S9). The test also determined that 98% of samples were correctly classified at the selected cut-off value that yielded maximum sensitivity and specificity (Figure S9). The sero-specificity of Rv1507A further suggests that it can be a suitable pathogen specific marker for diagnosis of active TB that can be used in tandem with host specific marker such as resistin (84–86) to design bimodal, rapid, and simple point of care (POC) diagnostic tests with enhanced ability to detect disease at early stages.

In line with these observations, we expected increased virulence in *M. smegmatis* when supplemented with Rv1507A. Our results suggested that Rv1507A knock-in *M. smegmatis* (Ms_Rv1507A) can survive for prolonged duration within macrophage. In response to infection by mycobacterium, macrophages mount physiological responses either in form of generation of ROS and reactive nitrogen species (RNS) to get rid of pathogen (87) or may undergo apoptosis in case it fails to get rid of pathogen (88). These results provide a mechanistic basis of induction of apoptosis in macrophages, infected with recombinant Ms_Rv1507A. Increased levels of ROS and NO leads to stress within the macrophages that result in apoptosis of macrophages. While cells undergo apoptosis to suppress replication of bacteria, pathogens such as *M. tb* try to inhibit apoptosis as a mechanism to subdue host immune response and can alternatively induce necrosis to disseminate infection to surrounding cells (89). Virulence and cellular apoptosis are two different aspects employed by bacteria for pathogenesis. While *M. tb* induces necrosis for dissemination, BCG and attenuated mutants including H_37_Ra predominantly induce apoptosis. Innate control of early bacterial growth is mediated through induction of apoptosis in infected macrophages which helps in dissemination of antigen reservoir from the cell. These antigens released from apoptotic cells allow cross priming in dendritic cells and activation of T cell mediated acquired immunity (90, 91). Apoptosis is considered as a host defense mechanism as it allows sequestration of pathogens within apoptotic vesicles leading to decreased bacterial viability (92).

In conclusion, we have shown the immunogenic and antigenic potential of *M. tb* specific Rv1507A protein that is absent in BCG vaccine. It elicits pro-host immune response under *in-vitro* or *in-vivo* conditions and can be further evaluated as a possible standalone subunit vaccine candidate. Though subunit vaccines are relatively safer than live vaccines but low immunogenicity and inadequacy of long-lived protection are areas of concern. The laborious process of purifying vaccine grade antigen multiple times and the use of adjuvant for enhanced immunogenicity that can potentially skew the immune responses are other potential limitations. Interestingly Rv1507A was inherently immunogenic, thus despite avoiding the use of adjuvant, it evoked substantial innate and adaptive immune response. On the contrary, higher magnitude of immune and memory response induced by Ms_Rv1507A compared to that of recombinant protein points to likely better efficacy of live vaccines. Live vaccines are highly immunogenic, and a single dose is usually enough for long term protection, but reversion to virulence is a serious limitation. Aerosol infection with Ms_Rv1507A reiterated that this recombinant vaccine is immunogenic but not virulent to cause any pathology. Further, the revival of BCG as a vaccine due to recent advancements in understanding of vaccine induced protection and the emerging role of trained immunity advocates use of Rv1507A for production of recombinant BCG vaccine. Integration of Rv1507A in BCG can elicit reinvigorate the missing central memory compartment that could sustain long term protection, missing in BCG. A similar study has already exhibited that RD 4 (Region of Difference 4) (Rv1501-1508c) that encompasses Rv1507A, when incorporated into BCG, demonstrated improved protection of zebra fish against *M. marinum* challenge than the parental BCG (93). Therefore, this pro-host antigenic protein could be a probable candidate to produce recombinant BCG with enhanced immune response. A lot of progress has been made to engineer BCG that has led to development of promising vaccines, however still considerable efforts are required to generate vaccines with functional long lasting memory.

## Data Availability Statement

All datasets generated for this study are included in the article/Supplementary Material.

## Ethics Statement

The studies involving human participants were reviewed and approved by Institutional Ethics Committee (IEC), NIP, New Delhi, India. The patients/participants provided their written informed consent to participate in this study. The animal study was reviewed and approved by Animal Ethics Committee, National Institute of Pathology (NIP), New Delhi, India.

## Author Contributions

NE, SR, and SH conceptualized and designed the research. SA, AA, and NN performed experiments. SA, AA, and JA carried out data analysis. SA, AA, NN, JS, SH, and NE wrote the manuscript. All authors contributed to the article and approved the submitted version.

## Conflict of Interest

SR was employed by the company BioInception Pvt. Ltd., United Kingdom. The remaining authors declare that the research was conducted in the absence of any commercial or financial relationships that could be construed as a potential conflict of interest.

## Supplementary Material

Figure S1 *In-silico* analysis of *M. tb* Rv1507A revealed its antigenic potential. **(A)** B-cell and T-cell epitope prediction in Rv1507A by IEDB tool (https://www.iedb.org/). Note the presence of multiple immunodominant epitopes that are allele independent suggesting global population coverage. **(B)** Also, note the absence of protein binding sites in disordered regions predicted using ANCHOR server (https://iupred2a.elte.hu).

Figure S2 *In-silico* analysis of Rv1507A and western blot analysis of *M. tb* culture filtrates revealed secretory nature of Rv1507A. **(A)** Localization prediction of Rv1507A by PredictProtein tool (https://www.predictprotein.org/) depicting its secretory nature. **(B)** Western blot image depicting the expression of Rv1507A in CFP of *M. tb* H_37_Rv. A distinct band is observed at 22KDa (WCL, Whole cell lysate; CFP, culture filtrate proteins; M, marker).

Figure S3 Expression and construction of Rv1507A knock-in *M. smegmatis*. **(A)** SDS-PAGE image depicting the elution of recombinant *M. tb* Rv1507A. A distinct band is observed at 22KDa (M: marker; L1: Lane 1; L2: Lane 2 FT: flow through; W1: Wash1; W2: Wash 2; E1-E5: Elutions). **(B)** Confirmation of recombinant *M. tb* 1507A using western blot. **(C)** Molecular characterization of pST-Ki_Rv1507A knock-in *M. smegmatis*. Confirmation of Sub-cloning of Rv1507A in pST-Ki expression vector using colony PCR (PC: positive control; M: marker; L: DNA ladder; 1-5: colony). **(D)** Confirmation of pST-Ki_Rv1507A in knock-in *M. smegmatis* using colony PCR. (PC: positive control; L: DNA ladder; 1–9: Colonies 1–9).

Figure S4 Ms_Rv1507A causes splenomegaly and increased number of splenocytes. Spleen was recovered from BALB/c mice (*n* = 6) that were injected with either PBS (uninfected) or Ms_Vc (1 × 107) or Ms_Rv1507A (1 × 107). **(A)** Representative picture of splenomegaly in the mice infected with Ms_Rv1507A as compared to Ms_Vc. **(B)** The number of splenocytes was counted after making single cell suspension of the spleen. Representative data show the number of splenocytes as mean ± SEM. Statistical significance was determined with one tailed Mann–Whitney test. *p* < 0.05 was considered significant, ^**^*p* < 0.01.

Figure S5 Ms_Rv1507A increases the expression of co-stimulatory markers in macrophage. RAW264.7 cells were infected with Ms_Vc and Ms_Rv1507A at an MOI of 10:1. Cells were harvested after 24 h of infection. The frequency of CD40^+^, CD80^+^, CD86^+^, MHCI, and MHCII markers was determined flow cytometrically. Representative data show the mean fluorescence intensity of cellular markers as mean ± SEM. Statistical significance was determined with one tailed Mann–Whitney test. *p* < 0.05 was considered significant, ^*^*p* < 0.05.

Figure S6 Rv1507A induces secretion of IFN-**γ** from re-stimulated splenocytes. BALB/c mice (*n* = 5) were immunized with purified recombinant Rv1507A proteins (10 μg/ml). Splenocytes (1 × 10^6^) isolated from mice were cultured in absence or presence of Rv1507A protein (2, 5, 10 μg/ml) for 48 h and the levels of IFN-γ were estimated by ELISA. Representative data show IFN-γ secretion as mean ± SEM. Statistical significance was determined with one tailed Mann–Whitney test. *p* < 0.05 was considered significant, ^***^*p* < 0.001.

Figure S7 Rv1507A knock-in *M. smegmatis* Ms_Rv1507A expresses Rv1507A. **(A)** Western blot confirmation of *M. tb* Rv1507A using in-house specific polyclonal antibody raised in rabbit. The different lanes are: Lane1: Protein molecular size marker; Lane 2 and Lane 3: Purified recombinant Rv1507A protein; Lane 4: Ms_Rv1507A knock-in *M. smegmatis* cell lysate; Lane 5: Ms_Vc knock-in *M. smegmatis* cell lysate. Note the presence of a band corresponding to 22KDa in lane 2, lane 3, lane 4, and absence in lane 5. **(B)** Growth curve of Ms_Rv1507A (black dots) as compared to vector control Ms_Vc (gray dots). Statistical significance was determined with two-way ANOVA. Note the absence of any significant difference in terms of growth kinetics between Ms_Rv1507A and Ms_Vc.

Figure S8 Rv1507A knock-in *M. smegmatis* exhibits increased survival in infected macrophages. RAW264.7 cells were co-cultured with SYTO-9 stained Ms_Rv1507A or Ms_Vc at MOI of 10:1. The uptake of Ms_Vc and Ms_Rv1507A cells within RAW264.7 macrophage cells were assessed by flow cytometry after 12, 24, and 48 h. Representative data from three experiments show mean fluorescent intensity (MFI) of fluorescently tagged viable Ms_Vc (black box) and Ms_Rv1507A (gray box) as mean± SEM. Statistical significance was determined with two-way ANOVA. *p* < 0.05 was considered significant, ^****^*p* < 0.0001.

Figure S9 Sensitivity and Specificity at various ODs. Highest value in correctly classified column was taken as cut-off, highlighted by blue enclosure.

Supplementary Table 1 Sequence of different primers used in the study.
