# Supplementary figures and images for: Immunodominant Mycobacterium tuberculosis Protein Rv1507A Elicits Th1 Response and Modulates Host Macrophage Effector Functions
Source: Front Immunol. 2020 Jul 21;11:1199. doi: 10.3389/fimmu.2020.01199 (PMC7385400; doi:10.3389/fimmu.2020.01199)

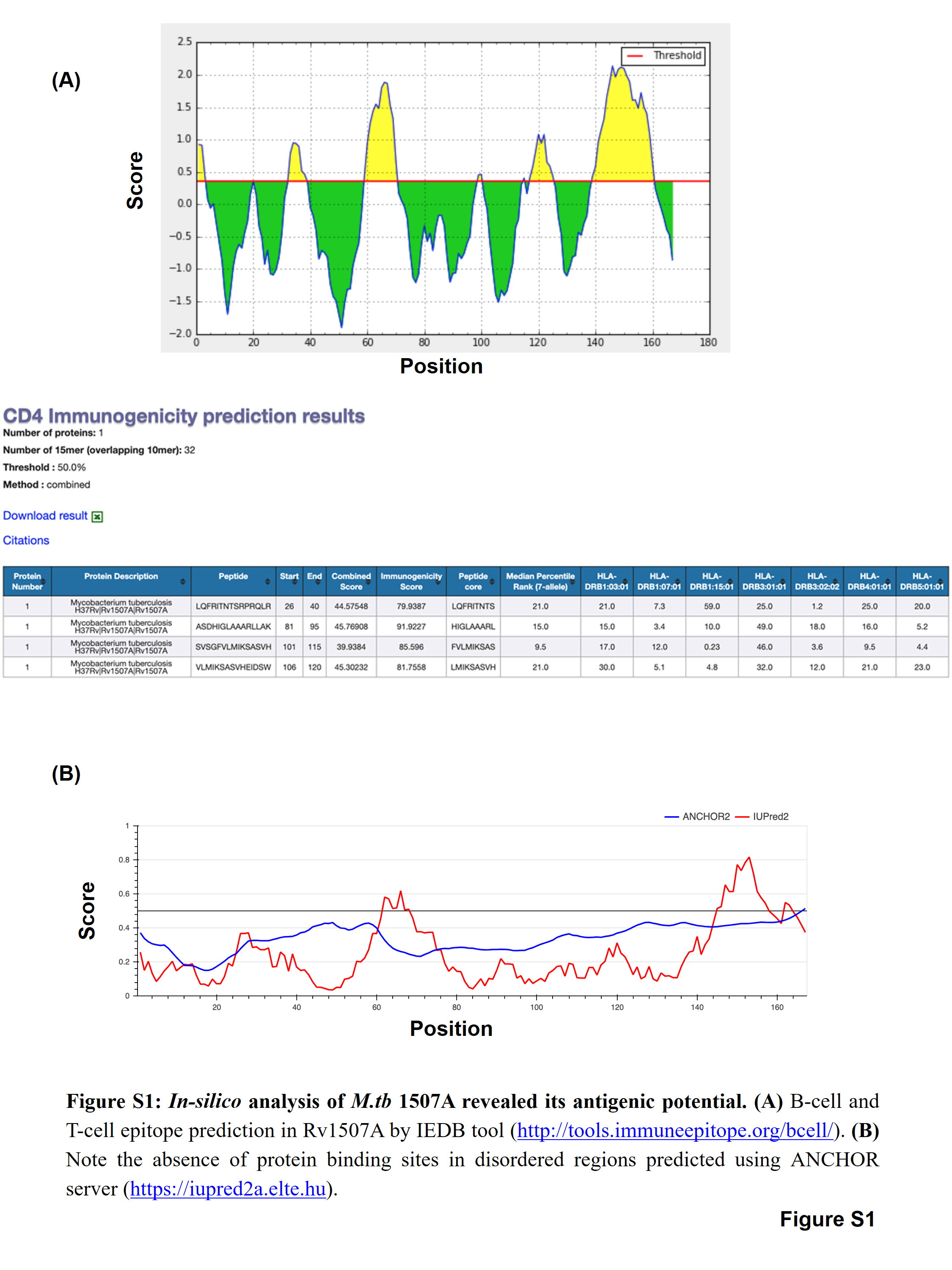

Supplement: Figure S1 — In-silico analysis of M. tb Rv1507A revealed its antigenic potential. (A) B-cell and T-cell epitope prediction in Rv1507A by IEDB tool (https://www.iedb.org/). Note the presence of multiple immunodominant epitopes that are allele independent suggesting global population coverage. (B) Also, note the absence of protein binding sites in disordered regions predicted using ANCHOR server (https://iupred2a.elte.hu). [file Image_1.TIF]

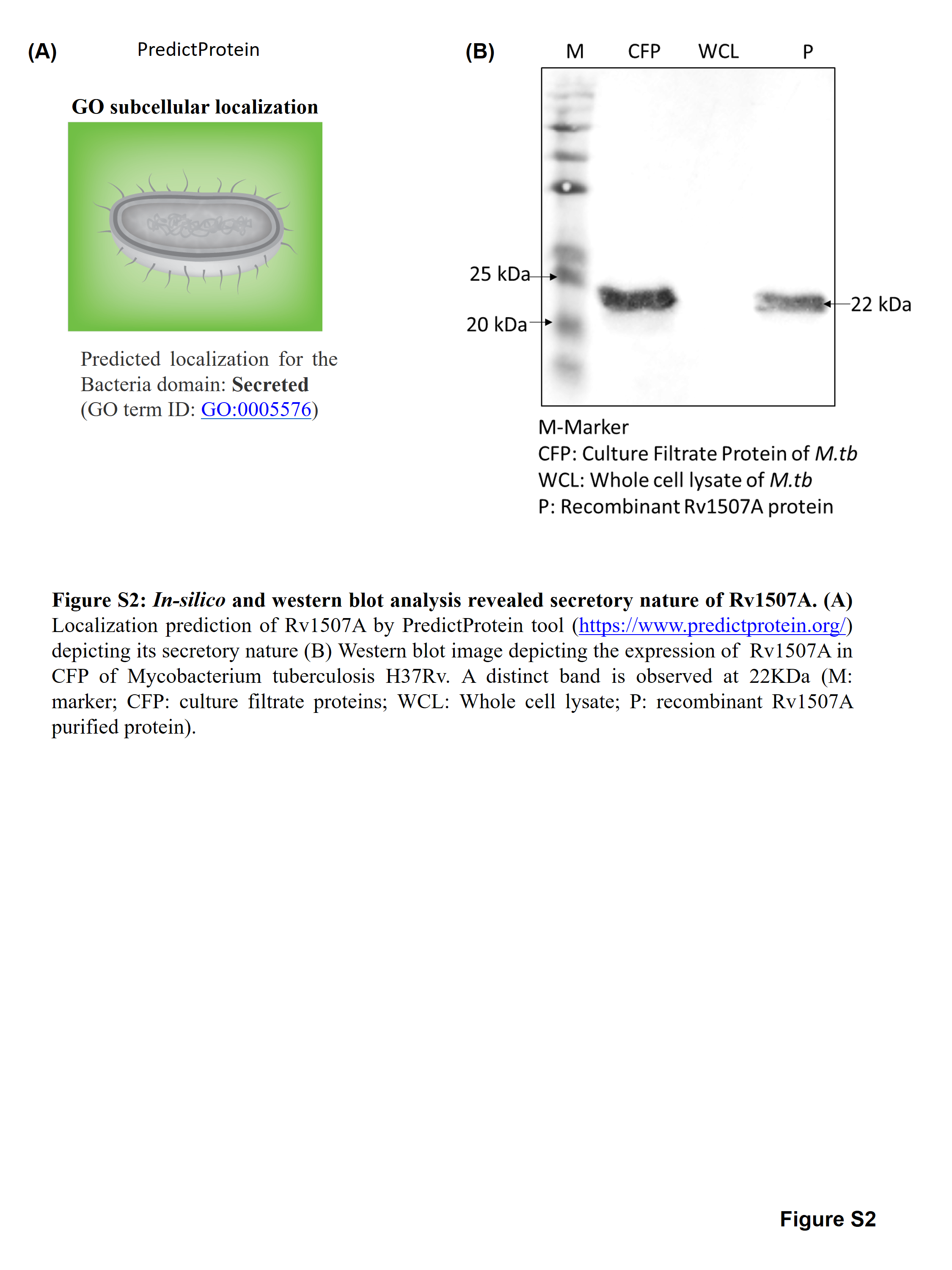

Supplement: Figure S2 — In-silico analysis of Rv1507A and western blot analysis of M. tb culture filtrates revealed secretory nature of Rv1507A. (A) Localization prediction of Rv1507A by PredictProtein tool (https://www.predictprotein.org/) depicting its secretory nature. (B) Western blot image depicting the expression of Rv1507A in CFP of M. tb H37Rv. A distinct band is observed at 22KDa (WCL, Whole cell lysate; CFP, culture filtrate proteins; M, marker). [file Image_2.TIF]

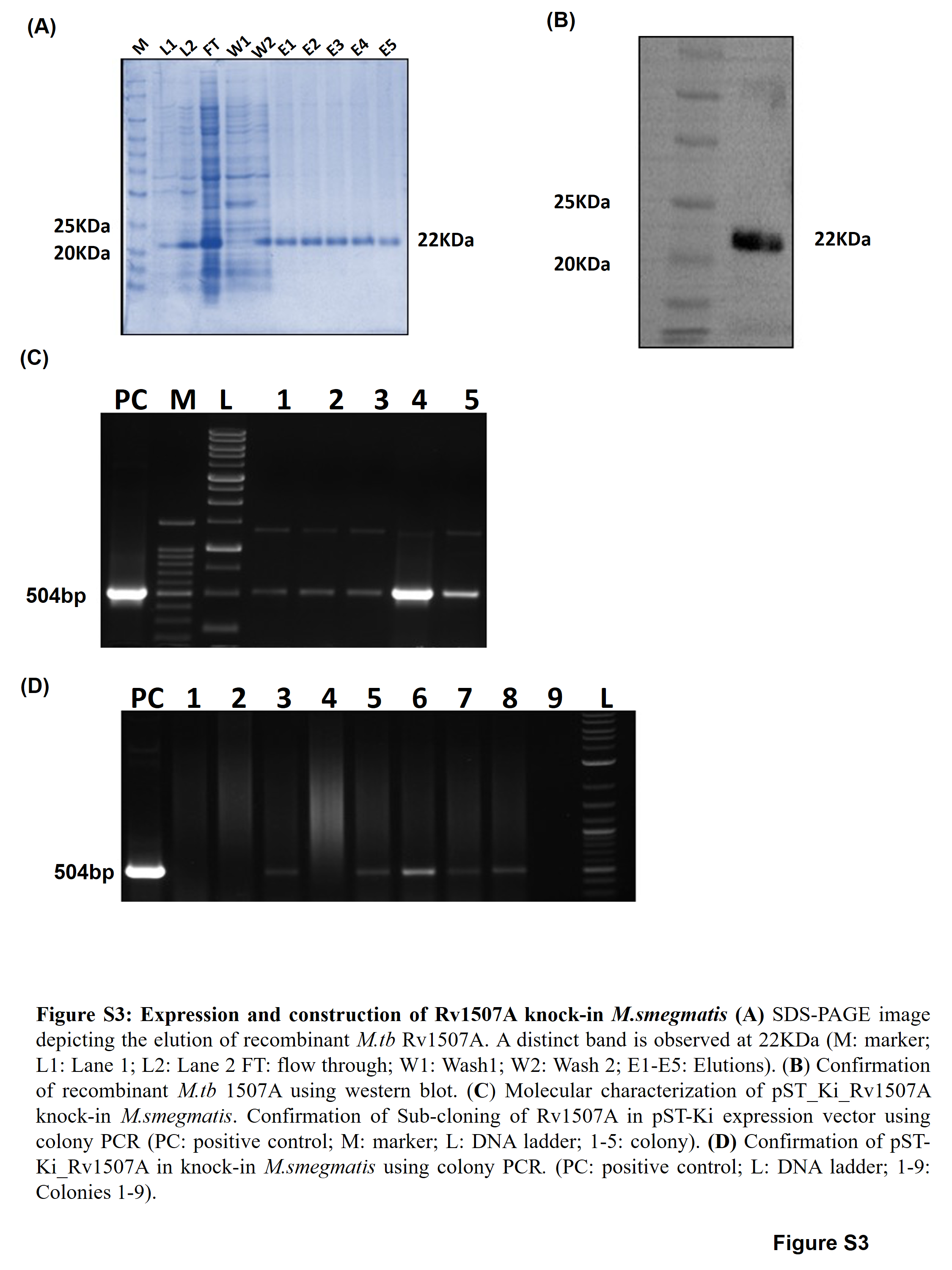

Supplement: Figure S3 — Expression and construction of Rv1507A knock-in M. smegmatis. (A) SDS-PAGE image depicting the elution of recombinant M. tb Rv1507A. A distinct band is observed at 22KDa (M: marker; L1: Lane 1; L2: Lane 2 FT: flow through; W1: Wash1; W2: Wash 2; E1-E5: Elutions). (B) Confirmation of recombinant M. tb 1507A using western blot. (C) Molecular characterization of pST-Ki_Rv1507A knock-in M. smegmatis. Confirmation of Sub-cloning of Rv1507A in pST-Ki expression vector using colony PCR (PC: positive control; M: marker; L: DNA ladder; 1-5: colony). (D) Confirmation of pST-Ki_Rv1507A in knock-in M. smegmatis using colony PCR. (PC: positive control; L: DNA ladder; 1–9: Colonies 1–9). [file Image_3.TIF]

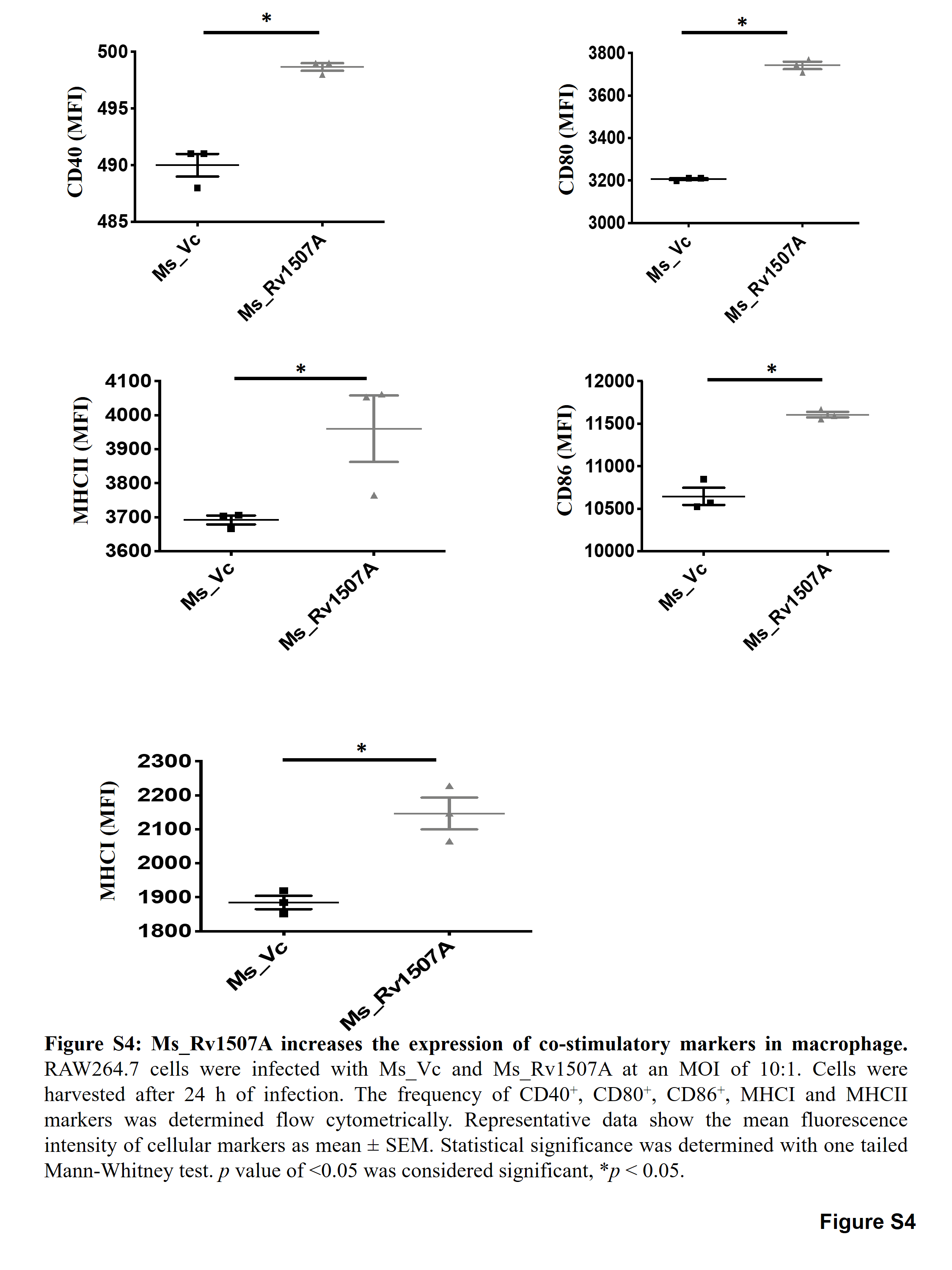

Supplement: Figure S4 — Ms_Rv1507A causes splenomegaly and increased number of splenocytes. Spleen was recovered from BALB/c mice (n = 6) that were injected with either PBS (uninfected) or Ms_Vc (1 × 10^7^) or Ms_Rv1507A (1 × 10^7^). (A) Representative picture of splenomegaly in the mice infected with Ms_Rv1507A as compared to Ms_Vc. (B) The number of splenocytes was counted after making single cell suspension of the spleen. Representative data show the number of splenocytes as mean ± SEM. Statistical significance was determined with one tailed Mann–Whitney test. p < 0.05 was considered significant, **p < 0.01. [file Image_4.TIF]

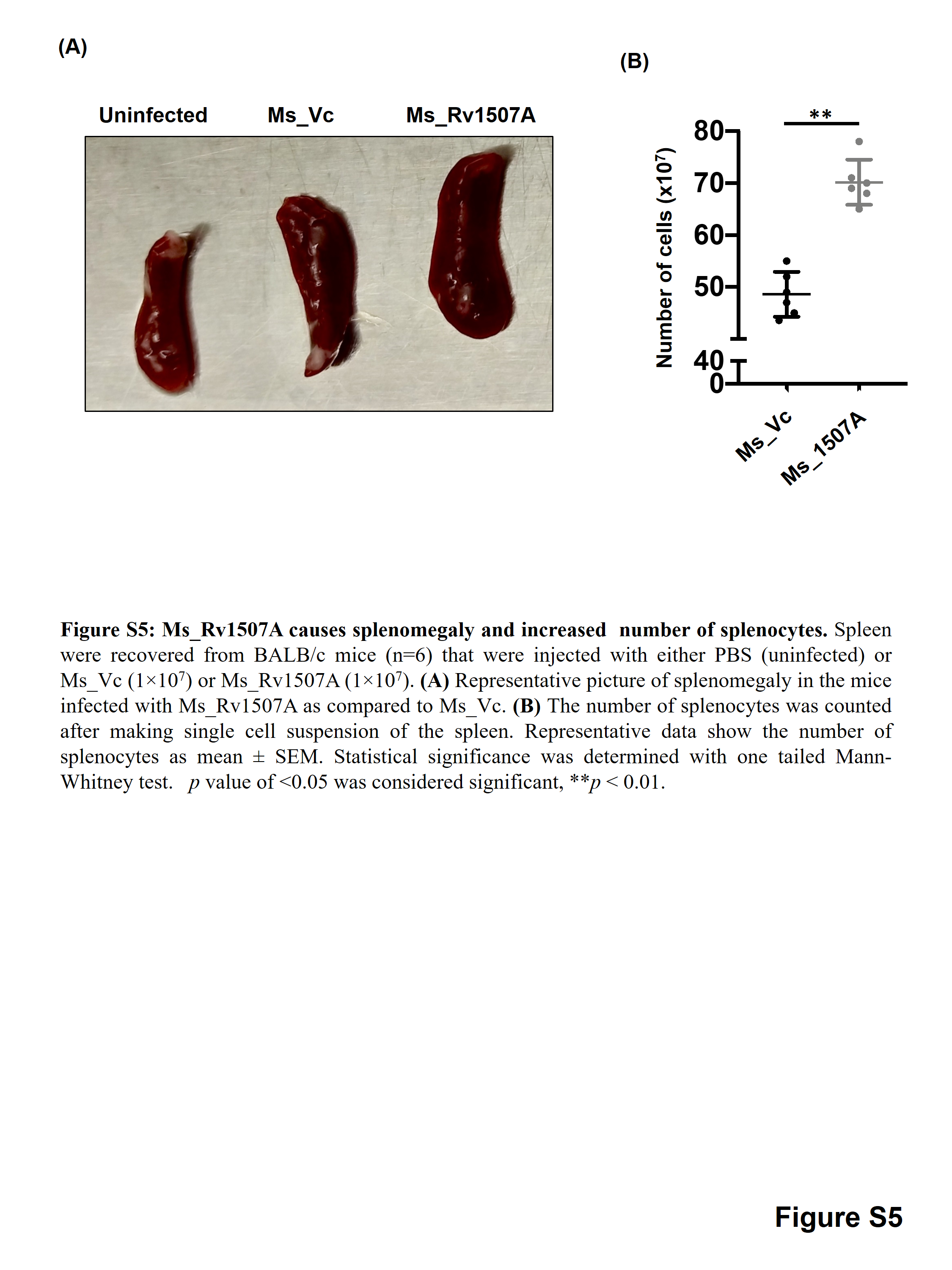

Supplement: Figure S5 — Ms_Rv1507A increases the expression of co-stimulatory markers in macrophage. RAW264.7 cells were infected with Ms_Vc and Ms_Rv1507A at an MOI of 10:1. Cells were harvested after 24 h of infection. The frequency of CD40+, CD80+, CD86+, MHCI, and MHCII markers was determined flow cytometrically. Representative data show the mean fluorescence intensity of cellular markers as mean ± SEM. Statistical significance was determined with one tailed Mann–Whitney test. p < 0.05 was considered significant, *p < 0.05. [file Image_5.TIF]

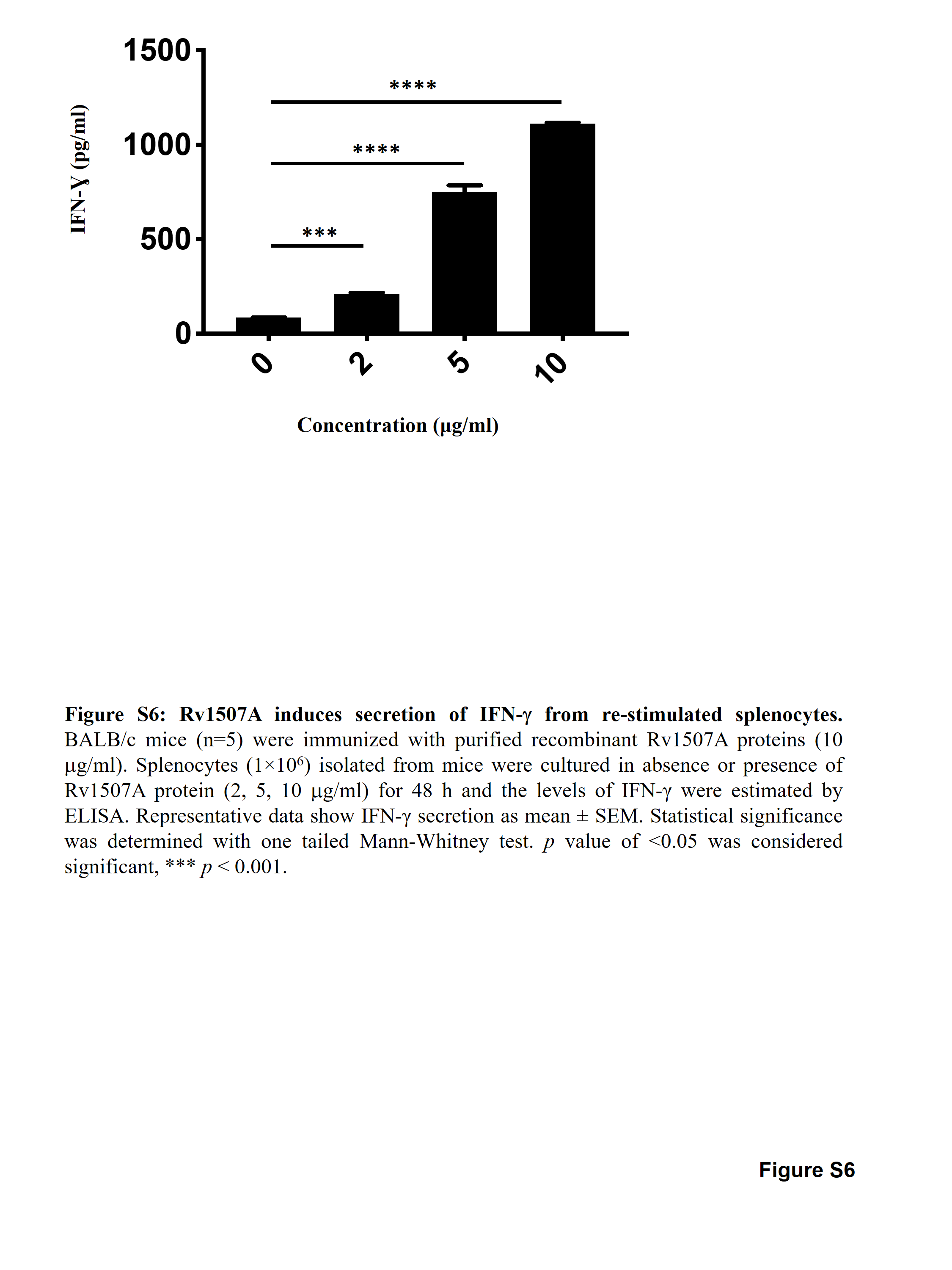

Supplement: Figure S6 — Rv1507A induces secretion of IFN-γ from re-stimulated splenocytes. BALB/c mice (n = 5) were immunized with purified recombinant Rv1507A proteins (10 μg/ml). Splenocytes (1 × 10^6^) isolated from mice were cultured in absence or presence of Rv1507A protein (2, 5, 10 μg/ml) for 48 h and the levels of IFN-γ were estimated by ELISA. Representative data show IFN-γ secretion as mean ± SEM. Statistical significance was determined with one tailed Mann–Whitney test. p < 0.05 was considered significant, ***p < 0.001. [file Image_6.TIF]

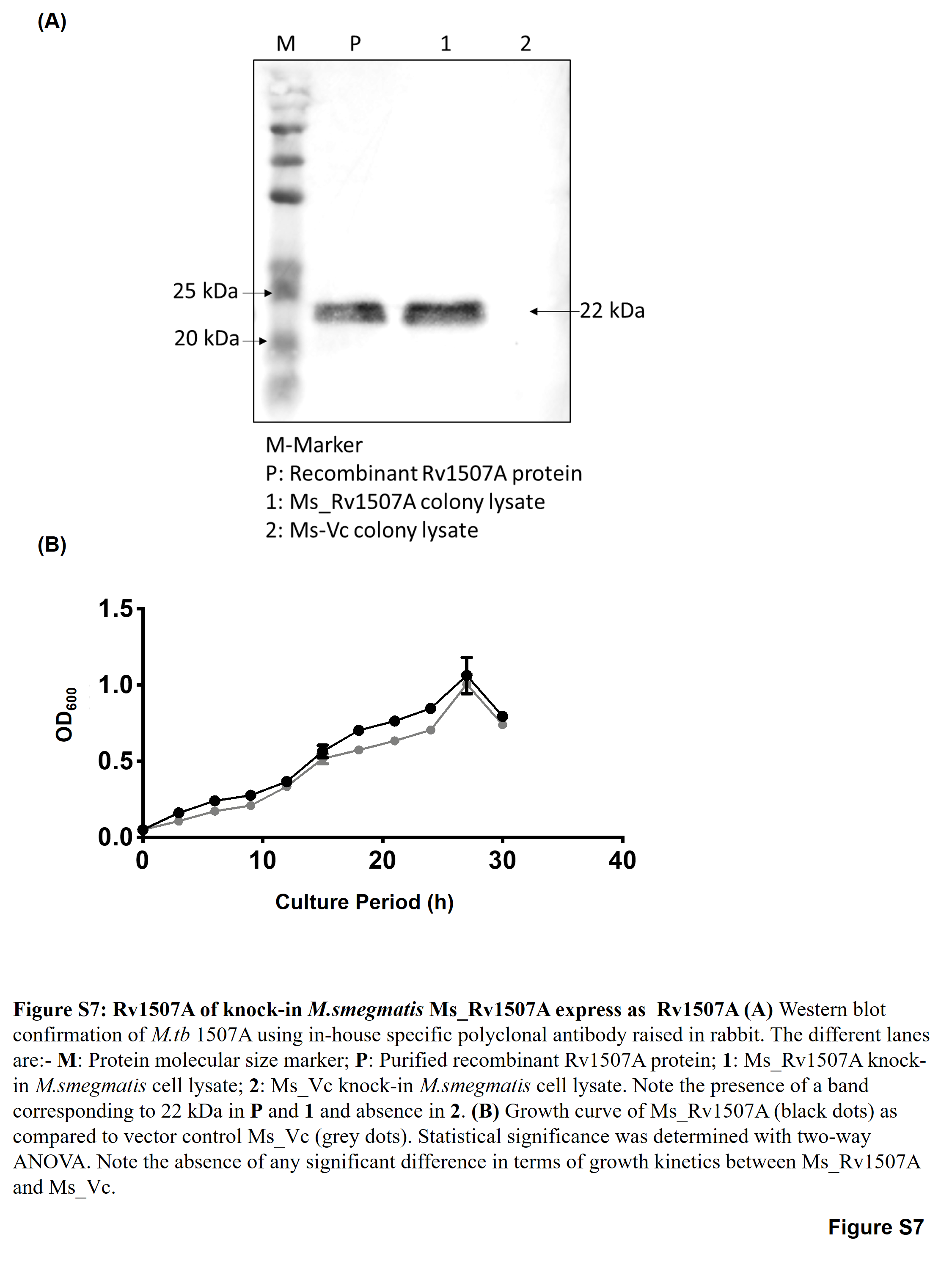

Supplement: Figure S7 — Rv1507A knock-in M. smegmatis Ms_Rv1507A expresses Rv1507A. (A) Western blot confirmation of M. tb Rv1507A using in-house specific polyclonal antibody raised in rabbit. The different lanes are: Lane1: Protein molecular size marker; Lane 2 and Lane 3: Purified recombinant Rv1507A protein; Lane 4: Ms_Rv1507A knock-in M. smegmatis cell lysate; Lane 5: Ms_Vc knock-in M. smegmatis cell lysate. Note the presence of a band corresponding to 22KDa in lane 2, lane 3, lane 4, and absence in lane 5. (B) Growth curve of Ms_Rv1507A (black dots) as compared to vector control Ms_Vc (gray dots). Statistical significance was determined with two-way ANOVA. Note the absence of any significant difference in terms of growth kinetics between Ms_Rv1507A and Ms_Vc. [file Image_7.TIF]

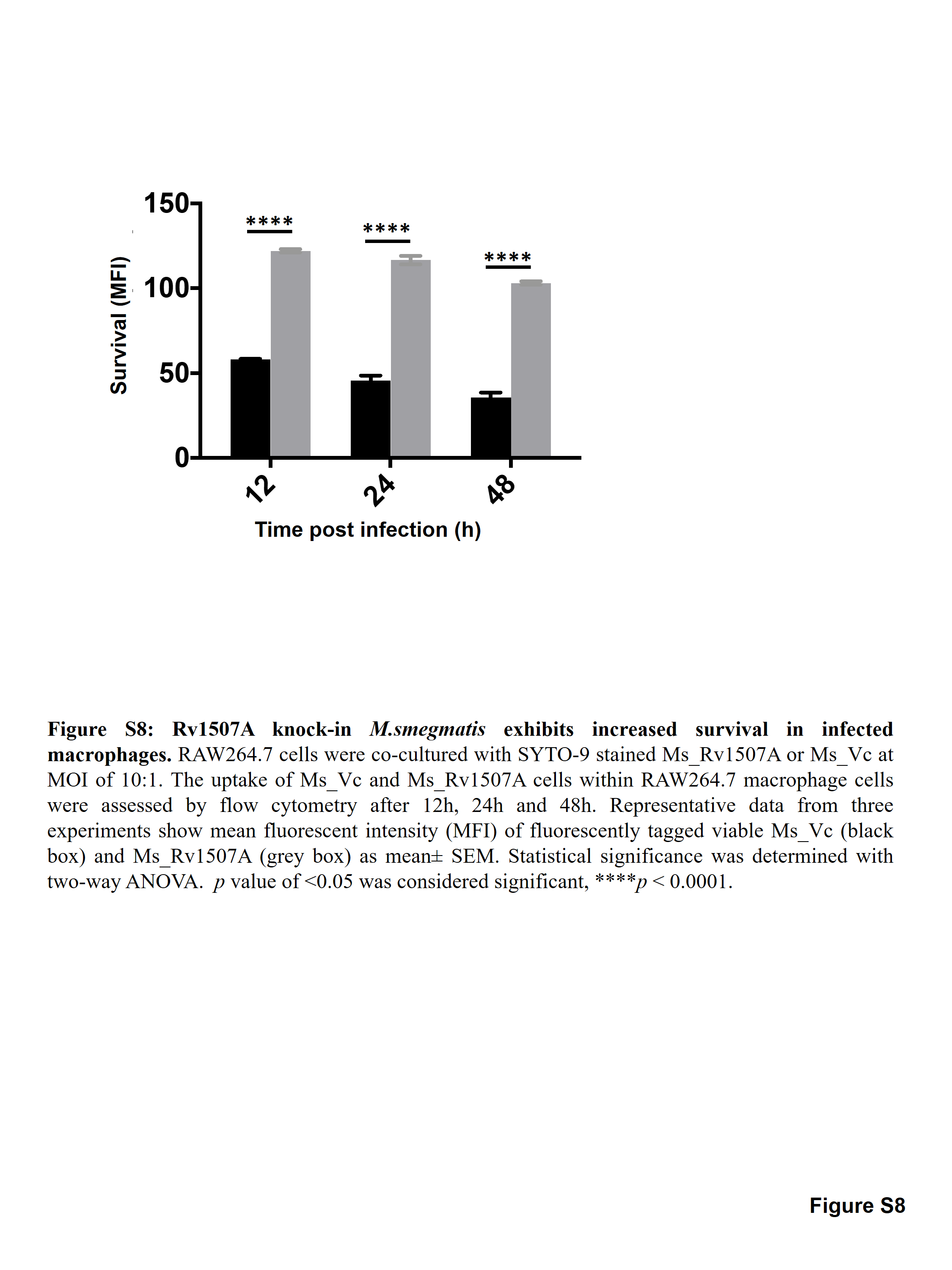

Supplement: Figure S8 — Rv1507A knock-in M. smegmatis exhibits increased survival in infected macrophages. RAW264.7 cells were co-cultured with SYTO-9 stained Ms_Rv1507A or Ms_Vc at MOI of 10:1. The uptake of Ms_Vc and Ms_Rv1507A cells within RAW264.7 macrophage cells were assessed by flow cytometry after 12, 24, and 48 h. Representative data from three experiments show mean fluorescent intensity (MFI) of fluorescently tagged viable Ms_Vc (black box) and Ms_Rv1507A (gray box) as mean± SEM. Statistical significance was determined with two-way ANOVA. p < 0.05 was considered significant, ****p < 0.0001. [file Image_8.TIF]

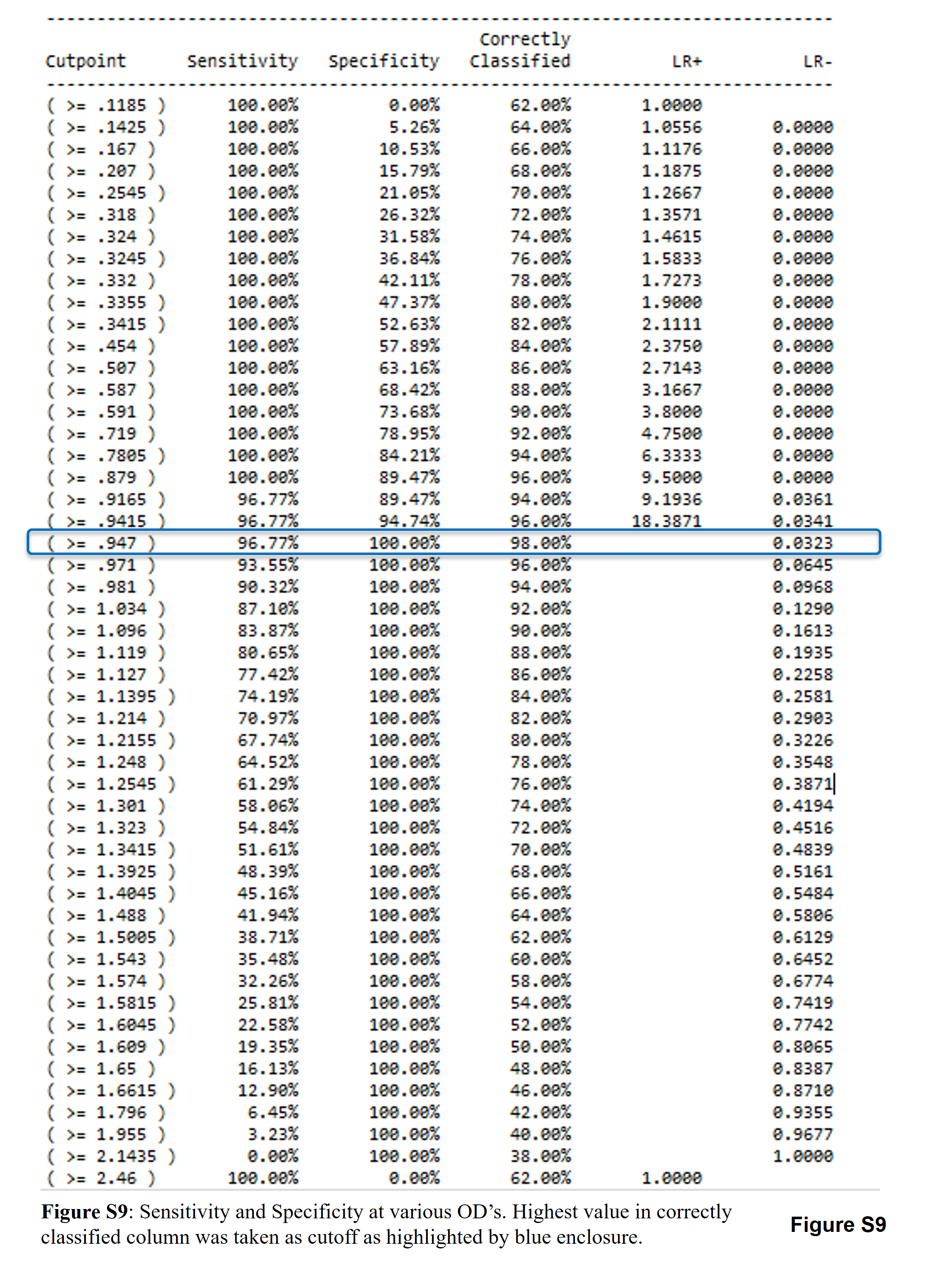

Supplement: Figure S9 — Sensitivity and Specificity at various ODs. Highest value in correctly classified column was taken as cut-off, highlighted by blue enclosure. [file Image_9.TIF]
