# Supplementary material for: Immunodominant Mycobacterium tuberculosis Protein Rv1507A Elicits Th1 Response and Modulates Host Macrophage Effector Functions
Source: Front Immunol. 2020 Jul 21;11:1199. doi: 10.3389/fimmu.2020.01199 (PMC7385400; doi:10.3389/fimmu.2020.01199)
Supplement: Supplementary Table 1 — Sequence of different primers used in the study. [file Table_1.docx]

**Supplementary Table 1: Sequence of different primers used in the study**

| **Primer** | **Sequence** | **Restriction enzyme site** |
| --- | --- | --- |
| Rv1507AF (P1) | ATGCAATCAGGTCAAAATATCCTCGCCA | EcoRI |
| Rv1507AR (P2) | ATCTCACAGGAGACGATGCGACGTTCAT | HindIII |
